# Transcription feedback dynamics in the wake of cytoplasmic mRNA degradation shutdown

**DOI:** 10.1093/nar/gkac411

**Published:** 2022-05-30

**Authors:** Alon Chappleboim, Daphna Joseph-Strauss, Omer Gershon, Nir Friedman

**Affiliations:** Alexander Silberman Institute of Life Science, Hebrew University of Jerusalem, Jerusalem 9190401, Israel; Rachel and Selim Benin School of Computer Science, Hebrew University of Jerusalem, Jerusalem 9190401, Israel; Alexander Silberman Institute of Life Science, Hebrew University of Jerusalem, Jerusalem 9190401, Israel; Rachel and Selim Benin School of Computer Science, Hebrew University of Jerusalem, Jerusalem 9190401, Israel; Alexander Silberman Institute of Life Science, Hebrew University of Jerusalem, Jerusalem 9190401, Israel; Rachel and Selim Benin School of Computer Science, Hebrew University of Jerusalem, Jerusalem 9190401, Israel; Alexander Silberman Institute of Life Science, Hebrew University of Jerusalem, Jerusalem 9190401, Israel; Rachel and Selim Benin School of Computer Science, Hebrew University of Jerusalem, Jerusalem 9190401, Israel

## Abstract

In the last decade, multiple studies demonstrated that cells maintain a balance of mRNA production and degradation, but the mechanisms by which cells implement this balance remain unknown. Here, we monitored cells’ total and recently-transcribed mRNA profiles immediately following an acute depletion of Xrn1—the main 5′-3′ mRNA exonuclease—which was previously implicated in balancing mRNA levels. We captured the detailed dynamics of the adaptation to rapid degradation of Xrn1 and observed a significant accumulation of mRNA, followed by a delayed global reduction in transcription and a gradual return to baseline mRNA levels. We found that this transcriptional response is not unique to Xrn1 depletion; rather, it is induced earlier when upstream factors in the 5′-3′ degradation pathway are perturbed. Our data suggest that the mRNA feedback mechanism monitors the accumulation of inputs to the 5′-3′ exonucleolytic pathway rather than its outputs.

## INTRODUCTION

Gene expression is a multistep process that starts at the nucleus, where structural (e.g. histones) and regulatory factors interact to facilitate transcription by the general transcription machinery and RNA polymerase II (PolII). During transcription, nascent mRNA molecules are capped at their 5′ end with a nucleolytic-resistant nucleotide (m7G), spliced, cleaved, and polyadenylated. Protein-mRNA complexes are then exported from the nucleus to undergo translation in the cytoplasm by ribosomes. To allow for dynamic gene expression, most mRNA species are actively degraded by cells within a short time frame (minutes in yeast to hours in mammalian cells (1,2)). mRNA degradation can be triggered by various quality control mechanisms (e.g. nonsense-mediated decay), but degradation is also thought to be coupled to the translation of the mRNA by ribosomes (3,4). Seminal work established the main degradation pathway in eukaryotes: mRNA is deadenylated by the Ccr4–Not complex, decapped by the Dcp1-Dcp2 complex, and degraded by the highly processive 5′-3′ exoribonuclease Xrn1 (5–10). An important alternate route involves the exosome, which degrades mRNA from its 3′ end following deadenylation by the Ccr4–Not complex. Additional less common endonucleolytic degradation pathways were also described (9,11).

As presented above, this process is largely unidirectional, namely, messages are generated in the nucleus, exported, translated, and degraded with no information flow back to the nucleus. However, mounting evidence from the past decade suggests that transcription in the nucleus is coupled to degradation in the cytoplasm. This coupling was demonstrated along two main branches of evidence. The first is gene-specific regulation of transcript fate by nuclear signals, e.g. replacing the promoter of a gene can alter its transcript half-life or cytoplasmic localization (12–16). In these cases, the functional implications are generally thought to be carried out by different mRNA-binding proteins that are exported with the transcript (17 ). Another line of evidence linking nuclear transcription and cytosolic degradation is a global phenomenon (termed mRNA buffering or mRNA homeostasis). It was demonstrated that large-scale perturbations to the degradation machinery are compensated by the transcription machinery (18–20) and vice versa (15,16,21–27). In these cases, the underlying mechanisms for the observed global transcription-degradation coupling remain contested and speculative. Proposed mechanisms involve several main components, including Rpb4/7 (POLR2D/G in humans) (27–29), Pab1/Nab2 (30–32), the Ccr4–Not complex (16,33), Snf1 (AMPK) (34,35), and Xrn1 (18,19).

Xrn1 is the main cytosolic 5′-3′ exonuclease in eukaryotic cells (6,8,36–38). Xrn1 knockdown causes developmental and fertility defects in multicellular organisms (39,40), and its knockout in yeast was shown to affect cell size and growth rate, hinder growth in certain stress conditions, and cause spindle-pole separation defects (41–43). In two important works published in 2013 Xrn1 was implicated in the coupling between degradation and transcription (18,19). However, these studies arrived at opposite conclusions about the role of Xrn1. Haimovich and colleagues reported that Xrn1 knockout maintains global mRNA levels. They could explain the observed buffering by attributing to Xrn1 (and related RNA binding factors) a role as a transcriptional activator that acts directly on chromatin. Conversely, in a systemic screen of RNA processing factors, Sun and colleagues reported that Xrn1 knockout results in the most significant increase in total mRNA levels, which is the result of a significant decrease in degradation rates and a slight increase in global transcription rates. Mechanistically, they linked Xrn1 levels to the transcript levels of a negative transcriptional regulator - Nrg1. Since then, other studies with Xrn1 knockout/knockdown exhibited various transcriptional effects (44–50). Similarly, the Ccr4–Not complex which is crucial for mRNA deadenylation and degradation was also implicated in transcription regulation (16,33,51,52). Interestingly, in the systemic screen by Sun et al. some components of the Ccr4–Not complex incur pronounced deviations from the wildtype strain (18).

To summarize, there is ample evidence that mRNA buffering takes place in perturbed yeast cells, and probably in higher eukaryotes, but there is little understanding of the mechanisms underlying various contradicting observations. We reasoned that if a feedback process is at play, the dynamics of the process can shed light on its mechanism. However, we found little information about the dynamics of the process, as most studies were performed in knockout strains at steady-state growth conditions. In several notable exceptions the time scale of the feedback was determined to be in the order of several minutes (23) and up to an hour (18,21), but these were observed in different settings, and their generality is unclear.

Several of the works mentioned here applied comparative Dynamic Transcriptome Analysis (cDTA (22)) to samples, which measures mRNA levels and monitors recently-transcribed molecules by pulse labeling RNA with uracil analogs. We adapted the cDTA protocol to a high-throughput, quantitative, and sequencing-based version that we termed cDTA-seq. The cDTA-seq technique allowed us to monitor Xrn1 depletion from cells in high resolution, and observe the dynamics of mRNA accumulation and cells’ adaptation to this perturbation. Utilizing metabolic labeling data we identify a delayed global reduction in transcription, which results in a return to wildtype mRNA levels after several hours. We further expanded our data to multiple other RNA processing factors and found that the transcriptional response was not unique to Xrn1, and it also occurred upon depletion of Dcp2 and Not1. Interestingly, we find that the transcriptional response initiates earlier when upstream components in the 5′-3′ pathway are perturbed, suggesting that the trigger for the transcriptional response is sensed upstream of the degradation pathway. These results provide a rich resource for studying the cellular response to perturbations in the general mRNA degradation machinery, and our analysis provides insights into the basic properties of the mechanism underpinning the mRNA buffering phenomenon.

## MATERIALS AND METHODS

### cDTA-Seq

#### Sample preparation

For each experimental batch, *Kluyveromyces lactis* (KL) yeast cells were grown to log-phase, fixed in frozen methanol and the KL-spiked methanol was equally pre-distributed in deep well plates in large volumes to minimize the effect of pipetting errors. The plate with pre-spiked methanol was kept at −80°C until the fixation of *Saccharomyces cerevisiae* (SC) samples. Immediately before SC fixation, sample biomass was measured by optical density at 600 nm and the culture was metabolically labeled by 4-thiouracil (4tU, Sigma). This was performed simultaneously on the entire plate using a pipetting robot (Tecan EVO200), after which cells were immediately fixed in pre-frozen and pre-KL-spiked methanol (600 μl pre-spiked frozen methanol to 500 μl SC cells). Samples were then kept at −80°C up to several weeks.

#### RNA purification

Cells fixed in frozen methanol were washed twice in ddw and RNA purification was performed as previously described (53) with minor modifications. Briefly, RNA was released from the cells by digestion with Proteinase K (Epicenter) in the presence of 1% SDS at 70°C. Cell debris and proteins were precipitated by centrifugation in the presence of potassium acetate. RNA was then purified from the supernatant using nucleic acid binding plates (96-well, 800 μl UNIFILTER Microplate, GE Healthcare) in the presence of 0.1 mM DTT, eluted in 1 mM DTT, and stored at −80°C.

#### Metabolic labeling, and adaptation of SLAM-seq to yeast

Metabolic labeling of recently transcribed RNA molecules was done as previously described (54,55). Briefly, 4tU was dissolved in NaOH and added to cells at a final concentration of 5 mM 4tU for the indicated times (6–10 min). To avoid pH change as a result of NaOH addition, MES buffer was added to the media prior to growth. RNA purification was performed as described above. Total RNA was subjected to thiol(SH)-linked alkylation by iodoacetamide (Sigma, 10 mM) at 50°C for 15 min, the reaction was stopped with 20 mM DTT. RNA was purified using nucleic acid binding plates (96-well, 800 μl UNIFILTER Microplate, GE Healthcare) and was stored with RNase-inhibitor at −80°C.

#### polyA RNA-seq library preparation

Library preparation was done as previously described (56). Total RNA was incubated with oligo-dT reverse transcription primers with a 7 bp barcode and 8 bp Unique Molecular Identifier (UMI) at 72°C for 3 min and transferred immediately to ice. RT reaction was performed with the SMARTScribe enzyme (Clontech) at 42°C for 1 h followed by enzyme inactivation at 70°C for 15 min. Barcoded cDNA samples were then pooled and purified using SPRI beads x1.2 (Agencourt AMPure XP, Beckman Coulter). DNA-RNA hybrids were tagmented using Tn5 transposase (loaded with oligos Tn5MEDS-A, Supplementary Table S2), and 0.2% SDS was added to strip off the Tn5 from the DNA (57), followed by a SPRI x2 cleanup. Barcoded Illumina adaptor sequences (Supplementary Table S2) were added to the tagmented DNA by PCR (KAPA HiFi HotStart ReadyMix, Kapa Biosystems, 12 cycles). And the DNA was cleaned with an x0.8 SPRI procedure. Libraries were sequenced using Illumina NextSeq-500 sequencer.

#### Data processing

##### Demultiplexing

Pooled libraries were demultiplexed using Illumina's bcl2fastq (version 2.20.0). Internal (sample) barcodes were demultiplexed with an awk command, not allowing any barcode errors in both cases.

##### Genome alignment

Prior to read alignment, we prepared several versions of the SC and KL genomes. First, we converted both genomes to accommodate alignments of partially (T→C)-converted reads. This was achieved by converting all the observed Ts in the genome to Cs per strand. This results in an ACG-only genome with one contig per reference strand. In addition, we also generated a redacted version of the KL genome in which any 18-mer that is found in the SC genome was removed. This procedure removed only ∼3.4% of the KL genome sequence while increasing the proportion of reads that uniquely align to the KL genome from ∼2.5% to ∼99% (i.e. most reads arise from regions that are shared between the genomes but are derived from SC cells).

polyA stretches were removed from 3′ ends, and reads with more than 25 bases remaining were aligned in several different ways (using default bowtie2 settings for single end alignment):

- SC genome without ACG conversion
- SC genome with ACG conversion
- KL genome without ACG conversion
- KL genome with ACG conversion
- Redacted KL genome without ACG conversion

These allow for quality control measures to be calculated per sample. However, for downstream analysis, only the (2) and (5) alignments are used. Alignment after ACG conversion was performed by converting observed Ts to Cs in reads and aligning them against the converted genome. Following alignment to the converted genome by bowtie2, a dedicated script converts reads back to the original reference coordinates and strand, and marks any sequence discrepancies between the original observed sequence and the reference sequence per read.

##### Read filtration and UMI handling

Reads in all sequencing runs had lengths between 44 and 46, and we discarded reads with >30 observed Ts (0.4% of reads). For typical analyses of mRNA, we only considered reads that were aligned to at most 5 genomic loci, with at most three errors (excluding T→C conversions). Out of these alignments, only the best one was reported. Reads were de-duplicated based on their alignment (chromosome, strand, position), and UMI, and in each such group, the read with least amount of deviations from the reference was selected. Libraries had an average of 1.2–1.5 reads per UMI.

##### Read statistics

Filtered and deduplicated alignments were assigned to transcripts by their intersection (58) with a window of 300 bp upstream and 100 bp downstream of previously reported transcription termination site (TTS) annotations (59). Read statistics were then collected on individual transcripts, groups of transcripts, or the whole transcriptome in the form of a table with the number of reads for each combination of observed Ts and observed T→C conversions.

##### Relative mRNA level estimation

To estimate the relative amount of mRNA in each sample we consider only the reads that were aligned to annotated TTS regions (see above). We divide this number by the sample OD, and by the total number of reads that were aligned to the KL redacted genome. While this procedure removes most of the variance between replicates, in time course experiments we also smooth these estimates with a savitzky-golay filter (3rd degree) spanning a 120-minute window.

##### Binomial mixture model

Similar to (60), we assume that reads arrive from a mixture of two kinds of molecules - old transcripts that were transcribed prior to the labeling period, and recent transcripts that were transcribed after the labeling period began. To estimate the typical molecule half-life, we are interested in the relative size of each of these sets. This proportion can be denoted with a single parameter—the recent fraction—${p_r}$. The model stipulates that if a molecule arrives from the ‘old’ set then we expect T→C conversions at a certain rate—$\varepsilon$, if however, the molecule is from the recent set, then we should observe conversions at a higher rate—$\xi$. In either case, assuming a uniform rate along the read, the probability of observing a read with X Ts, Y of which are converted is binomially distributed:

$$\begin{equation*}{O_{Y,X}} \equiv Pr(Y|X,old) \sim Bin\left( {Y;X, \varepsilon } \right)\end{equation*}$$

$$\begin{equation*}{R_{Y,X}} \equiv Pr(Y|X,new) \sim Bin\left( {Y; X, \xi } \right)\end{equation*}$$

Therefore, the overall probability of observing *Y given X* is:

$$\begin{equation*} Pr(Y|X) = p_rR_{Y,X} + (1-p_r)O_{Y,X} \end{equation*}$$

And more generally, the likelihood of a collection of reads, *R*:

$$\begin{eqnarray*}L\left( {{p_r}} \right) &=& Pr(R\ |\ {p_r}) \\ &=& \mathop \prod \limits_{r \in R}^{} {\left[ {{p_r}{R_{Y\left( r \right),X\left( r \right)}} + \left( {1 - {p_r}} \right){O_{Y\left( r \right),X\left( r \right)}}} \right]_{}}\end{eqnarray*}$$

Thus, assuming the error and conversion rates are global (Supplementary Figure S1E), the likelihood is a function of a single parameter—${p_r} \in [ {0,1} ]$, and its maximum can be efficiently calculated given the other model parameters (see supplementary material).

##### Steady-state model

We assume the following dynamic system:

$$\begin{equation*}\dot{M} = \ \pi C - \delta M\end{equation*}$$

$$\begin{equation*}\dot{C} = \ \gamma C\end{equation*}$$

where $M$ is the total amount of mRNA, C is the number of cells (or, alternatively this can be biomass), $\pi$ is a gene-specific transcription rate per cell, $\delta$ is the gene-specific degradation rate, and $\gamma$ is the growth rate. Redefining $R\ = \frac{M}{C}$ , i.e. mRNA/cell, the system simplifies to:

$$\begin{equation*}{\rm{\ }}\dot{R} = \ \pi - \left( {\delta + \gamma } \right)R\end{equation*}$$

We also assume that 4tU labeling does not disturb cells from their exponential growth pseudo-steady-state (Supplementary Figure S1G, H), in which case the steady state value of the system is given by:

$$\begin{equation*}{\rm{\ }}{R_{ss}} = \frac{\pi }{{\delta + \gamma }}\ \end{equation*}$$

Additionally, the differential equation yields the following dynamics for molecules transcribed during the labeling period ($N$):

$$\begin{equation*}N\left( t \right)\ = \frac{\pi }{{\delta + \gamma }}\ \left( {1 - {e^{ - t\left( {\delta + \gamma } \right)}}} \right)\end{equation*}$$

Thus, the proportion of labeled transcripts, *p_r_* , evolves with labeling time (*t*), as follows:

$$\begin{equation*}{p_r}\left( t \right)\ = \ \ \frac{{N\left( t \right)}}{{{R_{ss}}}} = \ 1 - {e^{ - t\left( {\delta + \gamma } \right)}}\end{equation*}$$

##### Half-life estimation

Briefly, we fit/measure several global parameters (detailed description is given in the supplementary material): incorporation probability ($\xi$), error probability ($\varepsilon$), conversion lag time (${t_0}$), and growth rate ($\gamma$). Once these are determined, the only gene-specific parameter is the degradation rate ($\delta$), which can be calculated by fitting the BMM model to the data to obtain *p_r_*, and by inverting the last equation we obtain the degradation rate:

$$\begin{equation*}\delta \ = \ - \frac{1}{t}ln(1 - {p_r}) - {\gamma ^{}}\end{equation*}$$

Which can be further transformed to a half-life ($ln( 2 )/\delta$).

### Auxin-induced degradation

In all experiments, yeast cells were grown in YPD at 30°C overnight, supplemented with a final concentration of 10 mM MES pH 6.0 buffer. 60 minutes prior to the beginning of the time course, samples with OD ranging from 0.3 to 0.6 were split to deep well plates and grown at 25°C with constant pipette mixing during the time course. At indicated times auxin (3-indolo acetic acid, Sigma) was added at a final concentration of 1–2.5 mM (Supplementary Table S4). Auxin stock was dissolved in DMSO to 2.5 M, and diluted 1:1 in 1 M NaOH before being added to samples to prevent sedimentation in the aqueous medium. When mock treatment was appropriate, the same amount of DMSO and NaOH was added to control samples.

### cDTA-seq calibrations

#### Spike-in titration

As a standard for mRNA levels in cDTA-seq experiments, we spike-in fixed amounts of exogenous cells to each sample. To evaluate this strategy, we performed a titration of spike-in cells (KL) in the 1%-5% range into an *SC* sample. SC and KL cells were grown to log-phase (od 0.5) and fixed separately in cold methanol (7.5 ml cells in 9 ml methanol). Fixed cells were mixed at varying ratios (1–5% KL) and were kept at −80°C. RNA purification and polyA RNA-seq libraries were prepared as described above.

#### Transcription inhibition

Thiolutin (Sigma) was dissolved in DMSO and added to cells at a final concentration of 3 μg/ml for 15 min.

#### DNA-seq validation

When comparing mRNA levels between samples, one can use different measures, such as mRNA/cell, mRNA/volume or mRNA/biomass. In our view, biomass or volume are the most relevant normalizing measures for the question of mRNA homeostasis, but cell counting remains common (22). In many experimental settings, the optical density (OD) of the culture is a good proxy for cell counts, but more generally OD quantifies the total sample biomass (which is correlated to cell counts (61)). In our experiments, there was a concern that different genetic backgrounds and cell states can affect the OD/cell ratio (62,63). We verified that our OD measures correspond to cell counts by comparing the DNA content extracted from 93 samples from various genetic backgrounds to their OD. To estimate the ratio between the number of SC and KL cells we extracted nucleic acids from various samples that underwent cDTA-seq and were therefore spiked-in with KL cells. These samples included various AID-tagged proteins (Dcp2, Xrn1, Rat1, Fcp1, Sth1, Med14, Pop2, Spt6) grown overnight with varying degrees of auxin, resulting in a wide range of growth rates (1.5–4 h doubling time) and morphological phenotypes (e.g. Xrn1-depleted cells are larger). When we compared the ratio between SC DNA and KL DNA in each sample with the OD of that sample, we observed a high correlation (*R*^2^ = 0.88, Supplementary Figure S1B).

To prepare DNA, RNase (Sigma, 11119915001) was added to nucleic acid extracted as mentioned in the cDTA-seq protocol (0.1 μl RNase to 100 ng RNA in a reaction volume of 20 μl) and incubated at 37°C for 30 min. The remaining nucleic acid - genomic DNA - was tagmented using Tn5 transposase (loaded with oligos Tn5MEDS-A and Tn5MEDS-B, Supplementary Table S2), and 0.2% SDS was added to strip off the Tn5 from the DNA (57). Barcoded Illumina adaptor sequences (Supplementary Table S2) were added to the tagmented DNA by PCR (KAPA HiFi HotStart ReadyMix, Kapa Biosystems, 20 cycles), and the libraries were cleaned with a ×0.9 SPRI procedure. Libraries were sequenced on an Illumina NextSeq-500. Reads aligned to the SC genome were manually inspected to have uniform genomic distribution (with exceptions in the rDNA locus, transposable elements, etc.). Reads were also aligned to a redacted SC genome (removing any 20-kmer found in KL) and to a similarly redacted KL genome to obtain estimates of the amount of DNA from each organism in the sample. The ratio between these numbers was compared to the OD of each sample (both measures were normalized to their median for visualization in Supplementary Figure S1B). Outliers were not of a specific strain, batch, or condition, pointing to measurement noise (probably in the OD), rather than inherent biases in the technique.

### Western blots

Yeast lysates were prepared as previously described (64) and proteins were analyzed using standard western blotting procedures with anti-FLAG M2 (Sigma F1804), and anti-Myc (Sigma M4439 clone 9E10).

### Growth assays and OD measurements

Optical density was collected using a Tecan Infinite F200 for 96-sample plates. Each well/sample was measured at five different positions and the median value was used as the OD measure. A background level was measured in each plate and was subtracted from the measured OD.

Growth assays were conducted using a Tecan Freedom Evo 2000 liquid handling station. The 200 μl 96 sample plate was incubated at 30°C, for >24 h with an automatic scheduled OD measurement in the Tecan Infinite F200 executed every hour (65).

A log-linear fit was applied to each consecutive set of 10 data points, and the minimal doubling time was determined by the fit with the highest slope among the fits that passed the 5% significance threshold (test for linear fit, Bonferroni-corrected).

### Microscopy

Samples along the depletion time course were fixed in 1% formaldehyde for 15 min and quenched in 125 mM Gly. Bright-field images were automatically collected in multiple fields per sample along an 8 μm z-stack (1μm step size) using a Scan^R system (Olympus). Cells were segmented using freely available yeast segmentation software (66).

### Single-molecule FISH

#### Sample preparation

In the first experiment (Figure 3), 120 ml Spt6^AID^ and 120 ml Xrn1^AID^ cells were grown to mid-log, and split. Half of the culture was supplemented with auxin (final 2.5 mM) and half with mock treatment (50% DMSO, 0.5M NaOH at equal volume). After one hour of incubation at room temperature samples were fixed in 5% formaldehyde and prepared as previously described (67) with fluorophore-conjugated (TAMRA or CAL Fluor Red 590) tiling probes for Msn2, Cln2, Cln3 and Suc2 (Biosearch technologies) were a gift from Naama Barkai and Jeffery Gerst (Msn2, Cln2, Cln3 sequences detailed in Supplementary Table S3, Suc2 as in (68)).

In the second experiment (Supplementary Figure S3), 40 ml of Xrn1^AID^ cells were grown to mid-log and split into four samples. Samples were supplemented with auxin so to have 240, 120, 60 and 0-min time points in an auxin time course. Samples were fixed and prepared with the probes for Msn2, Cln2, Suc2 (and with a TAMRA-20dT probe ordered from IDT, see polyA FACS below).

#### Microscopy

Images were acquired with a 100 × 1.4 oil UPLSAPO objective, using an Olympus IX83 based live-imaging system equipped with CSU-W1 spinning disc (sCMOS digital Scientific Grade Camera 4.2 MPixel, Oxford Instruments, Abingdon, UK). For each sample, at least 4 different positions were chosen. In each position, three-channel Z-stacks images were taken with a step size of 200 nm for a total of >8 μm: bright-field image, 488 nm laser with 100 mW; DAPI image, 405 nm laser with 120 mW, and exposure time of 250 ms; mRNA image, 561 nm laser with 100 mW and exposure time of 1000 ms. Each z-plane image was of size 2048 × 2048 pixels.

#### Image analysis

Nuclei and single molecules were segmented from the DAPI channel using a MATLAB script that uses basic image processing steps (erosion/dilation/convolution) to account for uneven illumination. Segmented nuclei were filtered by size and manually inspected. Single-molecule counts were obtained by using custom-made MATLAB software (69).

### polyA FACS analysis

Cells from the second FISH experiment were stained with a polyT-TAMRA probe. At least 7500 valid events (cells) were collected per sample on an Amnis CellStream high throughput flow cytometer after excitation with a 561 nm laser and acquisition with a 561–583 nm filter.

### DNA staining

Wildtype, Xrn1^AID^ and Sth1^AID^ cells were split and exposed to auxin along a time course of 4.5 h. At the end of the time course, cells were collected into pre-frozen EtOH (final 70%). Cells were washed in 50mM Tris–HCl pH 8 (Sigma), incubated with RNaseA (Sigma R4875, final 1 mg/ml in 50 mM Tris–HCl pH8), Proteinase K (Sigma P2308, final 2.5 mg/ml in ddw), and with SYBR green (Molecular Probes S7567, diluted 1:1000 in 10 mM Tris–HCl pH 8, 1 mM EDTA pH 8). Following an additional wash, cells were briefly sonicated and analyzed by FACS with a 525-centered filter (BD LSRII system, BD Biosciences). Cell-cycle phases were determined by fitting the DNA content distribution with a 3-component gaussian mixture model.

### Strains and plasmids

*Saccharomyces cerevisiae* yeast strains used in this study are provided in Supplementary Table S1. Validations of AID degradation are also detailed in this table (see also Supplementary Figure S8 for a summary of western blots).

Yeast strains were generated using the LiAc transformation method (70). Auxin inducible degradation domain was PCR-amplified from plasmid pNat-AID*-9MYC or pHyg-AID*-6FLAG using matching primers (Supplementary Table S2) and introduced into TIR1 expressing cells immediately before the target gene stop codon (plasmids and TIR1 cells were a gift from Ulrich lab (71)).

Fresh Xrn1 knockouts were prepared using plasmids pNat-AID*-9MYC or pHyg-AID*-6FLAG, and oligos (xrn1_ko_F, xrn1-deg-R, see Supplementary Table S2)

Oligos used in this study are listed in Supplementary Table S2.

## RESULTS

### High-throughput, quantitative dynamic transcriptome analysis by sequencing (cDTA-seq)

**Figure 1.**
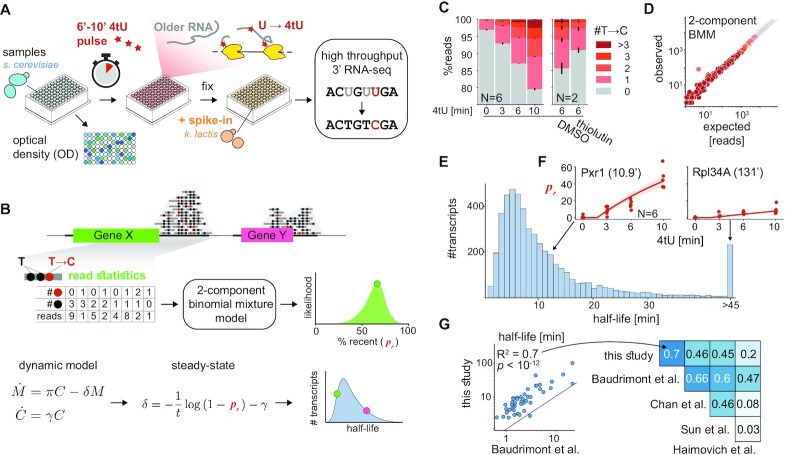
cDTA-seq and genome-wide transcript half-life estimation. (**A**) cDTA-seq protocol outline. 4tU is added to dozens of quantified samples to label new RNA molecules. Cells are then immediately fixed with a pre-fixed constant amount of spike-in cells (K. lactis yeast in this case). RNA is extracted, 4tU is alkylated and RNA-seq libraries are prepared, resulting in T→C conversions where 4tU was incorporated. The entire process is performed in a 96 sample format. (**B**) Transcript-level analysis outline. Read conversion statistics per gene are fitted with a binomial mixture model to estimate the percent of recently-transcribed molecules (p_r_). Assuming a first-order kinetic model (C - number of cells, *M*—number of mRNA molecules, $( {\gamma ,\pi ,\delta } )$ are growth, production, and degradation rates respectively), and assuming steady-state, p_r_ can be translated to transcript half-life given the known labeling period (t). See methods and supplementary material for more details. (**C**) 4tU conversion is effective, reproducible and measures transcription. Percent of reads (y-axis) along a 4tU time course (x-axis) with a different number of observed T→C conversions (legend, *N* = 6). Samples exposed to vehicle (DMSO) or the transcription inhibitor thiolutin for 15 min (and labeled with 4tU for 6 min, *N* = 2). Note that the y-axis begins at 75%, i.e. most reads have no conversions. (**D**) Binomial Mixture Model (BMM) fits the data. Read conversion statistics are fitted with a 2-component BMM. Each dot represents the number of reads with a certain number of observed Ts and T→C conversions (color as in E). x-axis is the expected number of reads for each (T,T→C) pair assuming the model and the observed #T distribution in the data, the y-axis is the observed number of reads in each (T, T→C) combination. Additional components do not improve the likelihood of the data (Supplementary Figure S1D). (**E**) Half-life distribution for all yeast transcripts. Assuming steady-state, transcript-specific and global parameters are iteratively fitted, resulting in the half-life of each transcript. The median of the distribution is 8.2', transcripts with a half-life of 45′ or longer are counted in the rightmost bin. (**F**) Examples of estimated *p_r_* along the time course. The individually estimated pr per time point and replicate (N = 6) for two transcripts (Pxr1 and Rpl34A) are shown as red dots along the time course. The data is fitted with a single parameter per gene (degradation rate) resulting in an estimate of 10.9’ half-life for Pxr1 and a 131’ half-life for Rpl34A. Using these estimates, the expected pr along the time course is plotted as a red line with 95% CI as a red shaded area. (G) Half-life estimates correlate with various studies. Half-lives from this and four other published studies are compared to each other and the linear explanatory value (R2) is denoted in the upper diagonal matrix. The scatter depicts a specific example of the comparison between this study and the rates from Baudrimont et al where estimates were not obtained by metabolic labeling.

To study the dynamic equilibrium of mRNA in cells a dynamic measurement is needed. We aimed to assess absolute mRNA levels and transcription rates in multiple strains in detailed time-course experiments, resulting in hundreds of samples. Therefore, we developed a protocol (Figure 1A) inspired by Sun *et al.* (22), in which we spike in a constant amount of cells from a close yeast species for mRNA quantification, combined with a brief pulse with 4-thiouracil (4tU) to quantify recently-transcribed molecules. Following similar works (54,55), we alkylate the 4tU nucleotides with iodoacetamide, which results in their subsequent reverse-transcription to G instead of A (T→C on the sense strand). The conversion allows for the detection of 4tU at the same time as quantifying mRNA levels with RNA-seq. Thus, we avoid additional biochemical separation and unknown losses in each sample, which allows for accurate estimation of the fraction of recently-transcribed molecules (Figure 1B). We incorporated these steps into an RNA extraction and 3′ mRNA sequencing protocol we had developed that was specifically aimed at high throughput measurements (56). The final protocol (Figure 1A-B) allows a single person to quantify the transcriptome, including recently-transcribed mRNA, from 192 samples in a single day (Materials and Methods).

To study changes to mRNA concentration, defined as molecules/biomass (see Materials and Methods), we needed two points of reference per sample - a measure of mRNA levels and of biomass. We verified our ability to quantify relative sample mRNA levels by spike-in titration (Supplementary Figure S1A, *R*^2^ = 0.99, *P* < 10^–13^). For biomass estimation, we measured optical density (OD), and we verified that there are no gross deviations between biomass and cell counts in the different genetic backgrounds we worked with (see methods, Supplementary Figure S1B, *R*^2^ = 0.88, *P* < 10^–47^).

Next, we performed a 4tU labeling time course experiment and observed a significant increase only in T→C conversions (Figure 1C, Supplementary Figure S1C). To verify that we can detect changes in transcription rates, we also performed this analysis after transcription inhibition with thiolutin for 15′ and pulse-labeled the samples with 4tU to observe a >50% reduction in labeled molecules (Figure 1C). As previously observed (60), individual reads exhibit multiple conversion events (Figure 1C) bolstering confidence that they arise from newly transcribed molecules. We used this observation to estimate the percent of molecules that were transcribed during the labeling period (‘*p*_*r*_’) by fitting a probabilistic model to the data (Figure 1B, methods). We verified that the model fitted the observed data well (Figure 1D, Supplementary Figure S1D), and that the data is best explained by an increase in the proportion of recently-transcribed molecules (*p*_*r*_), rather than changes to incorporation efficiency or other artifacts (Supplementary Figure S1E).

Assuming a steady-state and a first-order model for mRNA (Figure 1B, Supplementary Figure S1F–H), a half-life was calculated per transcript (Figure 1B, E, F, Supplementary Figure S1I, J). We compared our estimates with four published works that measured or estimated transcript half-lives using various methods (Figure 1G) (1,18,19,72). Our technique is clearly correlated to other studies, but there are global discrepancies between half-life estimates that were previously noted (Supplementary Figure S1K) (22,73,74), and are potentially explainable by technical discrepancies (e.g. maturation and polyadenylation time can cause an offset between techniques, see also Supplementary Figure S1L).

We conclude that cDTA-seq can be used to determine the relative mRNA level and estimate the half-life of individual transcripts from a single measurement, with the caveat that the absolute half-life numbers from any technique should be treated with care. Therefore, we include a wildtype sample in each experimental batch, which is used as a control for physiological anomalies and technical discrepancies between experiments.

### Turnover rates are slowed in the absence of Xrn1, but Global mRNA levels are maintained

**Figure 2.**
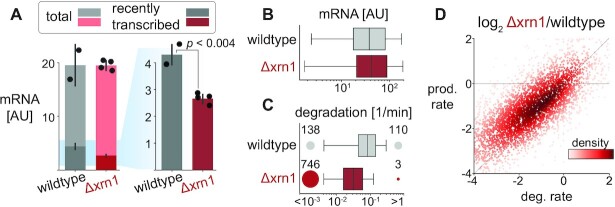
Xrn1 knockout causes a genome-wide decrease in degradation and transcription rates but maintains global mRNA levels. **(A)** Total mRNA levels are maintained but the recently-transcribed fraction decreases significantly. The amount of total mRNA (y-axis) in wildtype and Δxrn1 is the same (cumulative bars), while the fraction of recently-transcribed molecules decreases significantly (*t*-test *P* < 0.004, dark bars, 4tU labeling was performed for 9 min). (**B**) mRNA distribution is relatively unchanged between Xrn1 and wildtype. Transcript abundance distribution in wildtype (gray) and Δxrn1 (red). Boxes throughout the manuscript mark the interquartile range (IQR) with whiskers at 1.5 × IQR. (**C**) Xrn1 knockout causes a transcriptome-wide decrease in degradation rates. Transcript degradation rate distributions. Circles (and numbers) to the left and right of boxes correspond to the number of transcripts that are too stable (half-life >3 h, left) or too volatile (half-life < 1 min, right) to be estimated confidently. (**D**) Changes in degradation and production rates are correlated. Production rates are inferred from mRNA levels and estimated degradation rates (methods). Log_2_ fold changes between Δxrn1 and wildtype in production rate (x-axis) and degradation rate (y-axis) per transcript (dots, colored by their local density). Pearson *r* = 0.87, *P* < 10^–300^.

Previous studies found Xrn1 knockout to cause a global increase in mRNA levels per cell (18), or conversely, an unchanged level of mRNA (19,49). To address the question of absolute mRNA levels, we applied cDTA-seq to quantify the changes in mRNA levels in the absence of Xrn1 and the underlying mRNA dynamics. Given that Xrn1 is a major mRNA degradation factor, a knockout of Xrn1 is expected to cause an increase in total mRNA levels. However, using our protocol we do not observe any difference in global mRNA levels (normalized to biomass) when comparing freshly deleted Δxrn1 strains to wildtype cells (Figure 2A). Further, despite significant changes to ∼400 transcripts (Supplementary Figure S2A), the overall distribution of mRNA was unchanged (Figure 2B). We then turned to examine the changes to transcription and degradation rates in the absence of Xrn1. Contradicting reports in the literature argue that transcription slightly increases (18)) upon Xrn1 knockout, or is markedly reduced (19). In our data (two biological replicates in two fresh knockouts) we find a significant reduction in the fraction of recently-transcribed molecules from 22.8% to 14% (Figure 2A, *t*-test *P* < 0.004). Using our data, we were able to estimate the degradation and transcription rates for ∼4800 transcripts (>70% of genes). Unlike mRNA levels, we do observe a clear reduction in degradation rates (median decrease of 40%, with 746 transcripts becoming completely stable, Figure 2C). A corresponding reduction in transcription rates (median decrease of 70%, Supplementary Figure S2B) can be inferred from the mRNA levels and degradation rate estimates. When we examined the relationship between changes in production and degradation rates per transcript we found strong agreement, consistent with buffering (*r* = 0.87, *P* < 10^–300^, Figure 2D, Supplementary Figure S2C).

To better understand the global changes in degradation and transcription rates, we looked for functional signatures in ours and in published Δxrn1 transcriptome data (18,75,76). In these studies, there is no data pertaining to absolute mRNA levels, but strikingly, in all three published datasets, Δxrn1 exerted the most pronounced change to the mRNA profile relative to wildtype compared to all other knockouts (Supplementary Figure S2D), and these profiles were correlated (Supplementary Figure S2E). However, we could not identify robust common targets or consistent functional enrichments in the sets of up- and down-regulated genes in the knockout studies (Supplementary Figure S2F). Similarly, we found that in our data virtually only ribosomal protein genes and ribosome biogenesis genes were exceptional, pointing to indirect growth effects (Supplementary Figure S2G). Having excluded functional explanations, we tried using various gene/transcript features and sequence information to explain observed changes in mRNA levels or degradation and transcription rates. However, our models only explained a small fraction of the observed variance (Supplementary Figure S2H-J, supplementary note).

We conclude that cells maintain their global mRNA levels in the absence of Xrn1 by reducing global transcription rates, but the specific details underpinning the homeostasis are obscure in the knockout strain.

### Conditional Xrn1 depletion reveals a global but transient increase in mRNA levels

**Figure 3.**
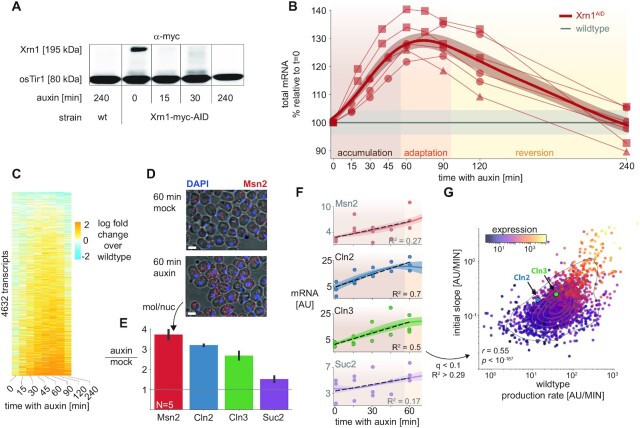
Xrn1 acute depletion causes a transient increase in mRNA levels. (**A**) Auxin inducible degradation (AID) of Xrn1 is rapid and stable. Western blot (anti-myc) for Xrn1 tagged with an auxin-inducible degron (AID) and a myc-tag. Shown are the isogenic untagged strain (left), and a time course that demonstrates virtually no Xrn1 protein within 15 min. osTir1 is also tagged with Myc in these strains and is used as a loading control. See also Supplementary Figure S5G. (**B**) Accumulation of mRNA immediately following Xrn1 depletion. mRNA counts (scaled by spike-in reads) from the Xrn1 depletion time course experiment (x-axis). mRNA is scaled to initial values, and to the corresponding wildtype measurement (y-axis) in a total of six biological replicates (lines) in three different experimental batches (markers). The gray line indicates the wildtype trajectory under the same transformation with standard deviation as a shaded area. The thick red line represents the average of the smoothed interpolations of each separate trajectory. We label the three stages of the response for convenience (accumulation → adaptation → reversion). (**C**) Genome-wide mRNA accumulation and reversion. mRNA per transcript (y-axis) was normalized with the corresponding measurement from the wildtype time course (time along the x-axis), and the log fold change is color-coded. Transcripts were filtered to not have any missing values along the trajectory (*N* = 4632, ∼70%), and were sorted by their average log fold change between 15 and 120 min. (**D**) Single-molecule FISH validation. Composite micrographs (from a confocal Z-stack image), showing DIC image of cells with a max-projection of DAPI stain in blue and fluorescent probes for Msn2 in red (1 μm scale bar). Individual molecules and nuclei are discernible and are counted. Xrn1^AID^ cells were treated with auxin or mock (DMSO) for 60 minutes, fixed, stained, and imaged. A clear increase in molecule counts is observed. (**E**) Single-molecule FISH quantification. In each field (N = 5), the number of observed molecules is divided by the number of observed nuclei to estimate the mRNA content of each cell in four different probes (x-axis). The y-axis denotes the ratio of the mRNA content in auxin versus mock treatment. (**F**) scaled RNA trajectories, and rate of accumulation. Each subplot shows the (spike-in scaled) mRNA counts (y-axis, points) from three replicates in the same experiment along the time course following Xrn1 depletion (x-axis). The line is the mean (+SEM) over interpolations of each separate repeat (*N* = 3). Selected transcripts correspond to smFISH probes (D, E). The dashed black line indicates the linear fit for the accumulation phase of the response (R^2^ noted per gene). Only fits within the 10% FDR threshold (*R*^2^ > 0.29, see S3E) are plotted in (G), Msn2 and Suc2 are shaded and do not appear in (G) as they are below this threshold. (**G**) mRNA accumulation correlates to transcript production rate. Comparing slopes from the FDR-selected linear fits (y-axis), to the production rate estimated from the wildtype sample (Figure 2D), 30% of the observed variability (R^2^) can be attributed to the production rate (*P* < 10^–187^). Note that there is a strong correlation between the two measures and the overall expression (color-coded, log scale), see text and Supplementary Figure S3F, G.

We reasoned that if Xrn1 is a major exonuclease of mRNA (6,18), its removal must exert an effect that is somehow buffered by cells. To test this hypothesis, we set out to repeat the cDTA-seq experiment in an Xrn1 conditional knockdown strain. We generated strains in which Xrn1 is tagged with an Auxin Inducible Degron (AID) (71,77) and validated that the Xrn1 protein is depleted rapidly from cells (half-life of ∼6', Figure 3A, Supplementary Figure S5G). To monitor the effects of Xrn1 depletion, we grew cells to an exponential growth phase and performed a 4-h time-course experiment following Xrn1 depletion (Figure 3B).

Multiple replicates reveal that mRNA levels increase significantly following Xrn1 depletion (20%-40% across multiple experiments and repeats; Figure 3B). However, after about 70 min from the time of auxin addition, the accumulation trajectory inverts, and we observe a decrease in global mRNA levels, resulting in a return to basal mRNA levels (90–105%) within 4 h. Importantly, the mRNA profile also converges to the Xrn1 knockout mRNA profile (Supplementary Figure S3A), validating the effectiveness of Xrn1 depletion. We call the initial time period (0∼55 min) the *accumulation* phase, the subsequent time (55∼95 min) the *adaptation* phase, and during the final *reversion* phase cells settle back to their initial mRNA levels (95 min and onwards).

An examination of the mRNA profile along the time course reveals that it is not the result of an increase in specific highly-expressed transcripts, but that virtually all transcript levels are transiently increased (Figure 3C). We validated the increase in mRNA levels following 60 min of mock or auxin treatment by single-molecule RNA-FISH with probes targeting four different transcripts and recapitulated the results from our spike-in-normalized mRNA-seq counts (Figure 3D, E, Supplementary Figure S3B). Notably, in a different time course FISH experiment, we observed that the reduction in the smFISH signal is slower than the observed reduction in the cDTA-seq signal, and we have evidence to suggest that this is a result of the previously reported accumulation of non-polyadenylated transcripts in Xrn1-depleted cells, which are not captured in the cDTA-seq protocol (Supplementary Figure S3C, D) (8).

These results highlight the ubiquitous nature of degradation by Xrn1 as evident by the immediate increase in the vast majority of transcripts and reveal the dynamics leading to homeostatic mRNA levels.

### mRNA accumulation correlates to transcription rates

While the response to Xrn1 depletion seems ubiquitous there are significant and reproducible differences between the response rate of different transcripts (Figure 3F). Given the direct nature of the perturbation, we wanted to test whether the observed changes to mRNA levels are consistent with a first order model for mRNA, whereby the immediate change in mRNA following a decrease in degradation is mainly a function of individual transcript production rate (see supplementary note). To test this prediction, we fit each transcript with a linear model for the change in mRNA during the accumulation phase of the response (Figure 3F). As predicted, the change in many transcripts is consistent with a linear increase (Supplementary Figure S3E, 10% FDR, *R*^2^ > 0.29, 2383/5164 transcripts), and where we cannot reject the null hypothesis of constant levels, it is mostly due to sampling noise (low expression levels, see for example Msn2 and Suc2 in Figure 3F). We tested the correlation of the fitted slopes to the pre-perturbation transcription and degradation rates and found that the strongest correlation is to the production rate, as expected by a first-order model (Pearson *r* = 0.55, *P* *<* 10^–187^, Figure 3G, Supplementary Figure S3F). To account for the potentially confounding effect of expression levels, which are correlated to both production rate and the slope (Figure 3G), we also calculated the partial correlation, which remained significant (partial Pearson *r* = 0.28, *P* < 10^–45^, Supplementary Figure S3F). Furthermore, this dependency is significantly accentuated in transcripts with a longer half-life (Supplementary Figure S3G), consistent with masking effects of residual degradation following Xrn1 depletion.

Therefore, during the accumulation phase, mRNA increase is consistent with a scenario where degradation is abruptly reduced, while transcription remains largely unchanged.

### Metabolic labeling through Xrn1 depletion uncovers a delayed global transcriptional adaptation

During the adaptation phase mRNA levels stop increasing and eventually revert to WT levels. How does this occur within a few hours? Since mRNA level is at a balance of transcription, degradation, and dilution by growth, there are multiple possible explanations.

Cells can adapt to reduced Xrn1-dependent degradation rates by activating alternative mRNA degradation mechanisms, increasing the dilution rate (which requires faster cell division and growth), decreasing transcription rates, or they can respond in some combination of these mechanisms.

We first tested the hypothesis that cells adapt by increasing their division rate or volume. We monitored various physiological aspects of cellular growth following Xrn1 depletion and found no significant inflection points in optical density, cell size, cell counts, colony-forming units, or cell-cycle fractions within two hours of auxin addition (Supplementary Figure S4A–E). Notably, some of these measures do eventually change, as previously reported for the knockout strain (e.g. increase in cell size, Supplementary Figure S4B). We also measured the growth rate in the absence of Xrn1 (knockouts and AID strains) and found a slower growth rate by 20–30% (Supplementary Figure S4F, G). We, therefore, exclude the possibility of volume or growth increase as possible explanations for the observed adaptation in mRNA levels within 2 h.

Another possibility is a compensatory increase in Xrn1-independent degradation. Xrn1 is the main 5′-3′ RNase in the cytoplasm, so the 3′-5′ degradation branch may be compensating for its absence. To test this hypothesis, we AID-tagged two components of the SKI complex (Ski2, Ski8) that were shown to be required for 3′-5′ degradation by the exosome (78) in addition to the Xrn1 AID tag. While these double-AID strains exhibited significantly slower growth when exposed to auxin (Supplementary Figure S4F, G), their immediate mRNA response to depletion was virtually identical to the Xrn1-AID strain (Supplementary Figure S4H), suggesting that the 3′-5′ degradation branch, as mediated by the SKI complex, does not take an active part in the observed reduction in mRNA levels.

**Figure 4.**
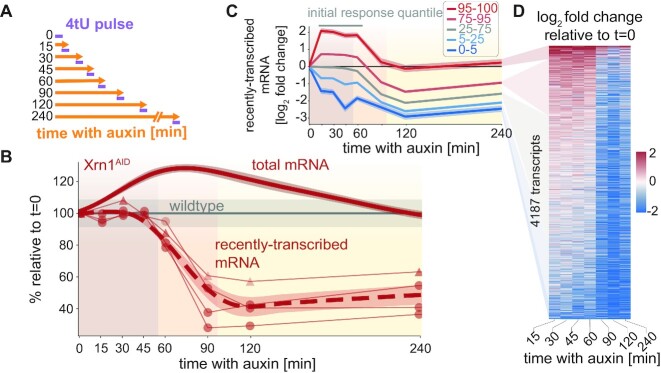
The transcription adaptation response to Xrn1 depletion. (**A**) AID/cDTA-seq experimental scheme. Cells are grown to the mid-log phase and split. At each indicated time-point auxin is added to an aliquot, and after 4 h (240 min) all are subjected to a short 4tU pulse simultaneously and harvested for cDTA-seq. (**B**) Recently-transcribed mRNA is reduced by ∼50% after ∼60 min. Global recently-transcribed counts (thin lines) relative to *t* = 0 from two experiments (markers) are plotted as a function of time since auxin addition (x-axis). The dashed red line represents the average of the smoothed interpolations of each separate trajectory (*N* = 4). The gray line indicates the wildtype trajectory under the same transformation with standard deviation as a shaded area. The solid red line is the change in total mRNA levels (same as in Figure 3B). (**C**) Transcription reduction is evident even in genes that were initially upregulated. Transcripts were grouped by their average initial change (15 < *t* < 60, gray bar above plot) into percentile groups (legend), and the average log fold change trajectory of each group (y-axis) was plotted as a function of time since auxin addition (x-axis). (**D**) Transcription reduction is abrupt and observed genome-wide. Color-coded log-fold change in recently-transcribed mRNA relative to *t* = 0 per transcript (y-axis) along the Xrn1 depletion time course (x-axis, excluding *t* = 0). Transcripts were filtered to not have any missing values along the trajectory (*N* = 4187, ∼63%), and were sorted by their average log fold change between 15 and 60 min.

To test the remaining possibility of transcriptional reduction, we examined the 4tU-labeled data obtained from cDTA-seq following Xrn1 depletion (Figure 4A). During the adaptation phase (55′–95′ following Xrn1 depletion) we observed a concerted and significant reduction in transcription (Figure 4B–D). Indeed, even genes that were induced immediately following Xrn1 depletion show a significant decrease at this point (Figure 4C, D), suggesting global repression of transcription, which we term the *transcription adaptation response*.

### Transient mRNA accumulation is recapitulated but dampened when upstream factors along the 5′-3′ degradation pathway are depleted

**Figure 5.**
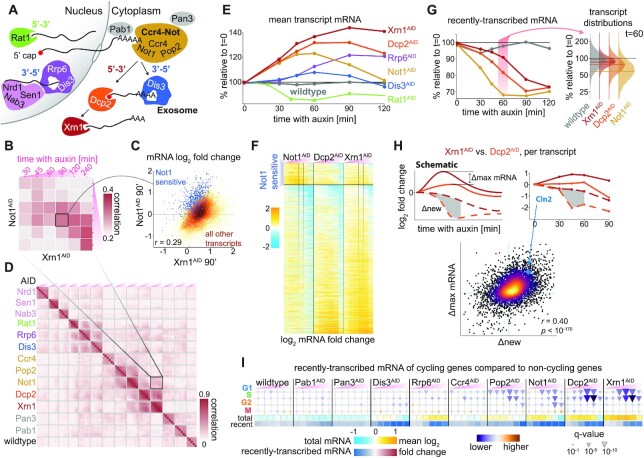
The transcription adaptation response is induced earlier when the 5′-3′ pathway is perturbed upstream. (**A**) AID-tagged proteins in this figure. Nuclear factors to the left - Rat1 is the nuclear 5′-3′ exoribonuclease, Nrd1, Sen1 and Nab3 survey aberrant transcripts and recruit the nuclear exosome (Dis3, Rrp6). Mature mRNA leaves the nucleus and will be deadenylated in the cytoplasm by the Ccr4–Not complex (Not1, Pop2, Ccr4). After deadenylation transcripts will continue to degrade 3′-5′ by the cytosolic exosome (Dis3), or 5′-3′ by Xrn1 after decapping by the DCP complex (Dcp2). Pab1 is the polyA binding protein, and Pan3 is part of an alternative deadenylation complex (37). (**B**) Factors noted in (A) were subjected to a 4-h depletion time course and cDTA-seq (as in Figure 4A). The matrix summarizes the Pearson correlation between the log-fold changes to transcripts relative to *t* = 0 in Xrn1 and Not1 depletion. Highlighted square corresponds to the scatterplot shown in (C). **(C)** Correlation between Xrn1 and Not1 depletion. Fold change relative to *t* = 0 after 90 min in Xrn1^AID^ (x-axis) and Not1^AID^ (y-axis). Changes are generally correlated. Set of Not1-sensitive transcripts are marked in blue, other points are colored by density. Data correspond to marked columns in (F) and marked square in (B). (**D**) Same as in (B), but comparing all time-course experiments. Highlighted square corresponds to the square depicted in (B). Note that the color scale is different. (**E**) Average changes to transcripts’ mRNA relative to *t* = 0 (y-axis) along the time course (x-axis) for factors exhibiting significant correlation to Xrn1 (Supplementary Figure S5A, see Supplementary Figure S5B for the 240’ timepoint). (**F**) mRNA changes upon interference to the 5′-3′ cytosolic pathway are correlated. Changes to transcripts (rows) along the time-course (x-axis, 15, 30, 45, 60, 90, 120, 240 min) in three time-courses: upon depletion of Xrn1, Dcp2, and Not1. The mRNA log fold change relative to *t* = 0 is color-coded. Transcripts are split into to Not1-sensitive (*N* = 672), and the rest of the transcripts (*N* = 4539). Rows in each set are sorted by the extreme point in a smoothed trajectory of the Xrn1 response. Highlighted columns correspond to the x- and the y-axis in (C). (**G**) The transcription adaptation response occurs earlier when the 5′-3′ pathway is perturbed upstream. Each line represents the average change (over all transcripts) to recently-transcribed mRNA relative to *t* = 0 (y-axis) along the depletion time course (x-axis) in each one of the four strains. The transcript-change distribution in each strain at the 60’ time point is detailed on the right, where the median of each distribution is denoted by a horizontal line. (**H**) Recently-transcribed mRNA differences explain total mRNA differences between strains. To compare the differences in response profiles between strains (in this case, comparing Xrn1 and Dcp2), we calculate the difference between the maximum observed change in mRNA per transcript (‘Δmax’, y-axis in scatter) and between cumulative nascent trajectories (shaded gray area in examples, ‘Δnew’, x-axis in scatter). We plot these statistics per transcript (dots in scatter, color denotes density) and found a significant correlation (Pearson *r =* 0.4 *P* < 10^–170^). See Supplementary Figure S5H for the same comparison between Dcp2 and Not1 (Pearson *r =* 0.31, *P* < 10^–98^). (**I**) A unique cell-cycle signature when the 5′-3′ degradation pathway is perturbed. We compared the distributions of transcription changes (relative to t = 0) in cell-cycle genes (rows) to the distributions of non-cycling genes (84). Significant deviations are denoted as colored triangles (purple/down—lower than non-cycling genes, orange/up—higher than non-cycling genes, size proportional to Kolmogorov–Smirnov *q*-value). Each triangle denotes the difference in a specific depletion time point (columns, x-axis time since auxin addition, same as in (B)). The bottom panels denote the average log fold change to mRNA and nascent mRNA in the same samples (same as in E and G). For further details, data, and analysis see supplementary material.

Xrn1’s function is largely attributed to 5′-3′ degradation after deadenylation and decapping (Figure 5A). We reasoned that we could pinpoint the molecular constituent sensed by cells by perturbing other factors in the mRNA degradation network and studying the changes to the transcription adaptation response. Therefore, we AID-tagged multiple components of this intricate network—Not1 (Cdc39), Dis3 (Rrp44), Rrp6, Rat1, Pop2, Pab1, Dcp2, Pan3, Nrd1, Sen1, Nab3 and Ccr4 (Figure 5A). We performed a depletion time-course experiment in these strains and applied cDTA-seq to hundreds of samples.

The changes in mRNA in this large dataset revealed the compartment, pathway, and protein-complex interactions between the depleted factors. For example, the effect of depletions of Rrp6, and Dis3—subunits of the nuclear and core exosome respectively (79)—are significantly correlated as expected, but are also correlated with depletion of other nuclear proteins (Figure 5B,D). We wanted to further dissect the response to Xrn1 depletion and focused on Xrn1-correlated factors - Rat1, Rrp6, Dis3, Not1, Dcp2 (Supplementary Figure S5A). We examined the overall mRNA profile following the depletion of these five additional targets (Figure 5E, Supplementary Figure S5B), and it became clear that responses upon Dcp2 and Not1 depletion were significantly more similar to Xrn1 depletion than all other ones (Supplementary Figure S5A).

Not1 and Dcp2 act upstream to Xrn1 in the 5′-3′ mRNA degradation pathway - Dcp2 is the catalytic component of the main decapping complex, and Not1 is the (essential) scaffold of the Ccr4–Not deadenylation complex. In all three depletion time courses (Xrn1, Dcp2, Not1) we observed an accumulation of mRNA followed by a reduction in mRNA levels, consistent with a general feedback mechanism that is triggered when this pathway is perturbed (Figure 5E, F). We compared the transcript profiles along the depletion time course (Figure 5F) and observed an overall high correlation between the perturbations (10^–300^*< P* *<* 10^–63^, Supplementary Figure S5A). More specifically it seems that mRNA accumulation is most pronounced in the case of Xrn1 depletion, slightly dampened when Dcp2 is depleted, and further muted when Not1 is depleted (Figure 5E, F).

Further examination of the data revealed a subset of ∼13% of transcripts that were more sensitive to Not1 depletion (Figure 5C, F). Functional analysis of these transcripts shows a strong enrichment for transcripts of proteolysis-related genes (*q* < 10^–8^, Supplementary Figure S5E). A link between Not1 and proteasome transcript regulation was reported in the literature, and recently, a co-translational complex assembly mechanism was suggested to be mediated by Not1 (80–83). The rapid and prominent increase in these transcripts, largely without a concomitant increase in transcription (Supplementary Figure S5F) suggests that these transcripts are especially susceptible to Ccr4–Not-dependent degradation directly or via the 3′-5′ degradation pathway. These results expand the previously reported link between Ccr4–Not and post-transcriptional regulation of the proteasome. Importantly, even in the Ccr4–Not-sensitive transcript cluster, mRNA accumulation following depletion of Xrn1 and Dcp2 is consistent with the global response pattern, suggesting that when Not1 is depleted a part of the observable increase in this cluster is due to accumulation emanating from the interference to the 5′-3′ degradation branch (Figure 5C,F, Supplementary Figure S5C, D).

These results demonstrate that mRNA generally accumulates in the same pattern when factors along the 5′-3′ degradation branch are perturbed, but the degree of accumulation depends on the specific element depleted (Not1 < Dcp2 < Xrn1).

### Upstream perturbations in the 5′-3′ degradation pathway result in earlier onset of the transcription adaptation response

To understand the apparent association between the global mRNA accumulation profile and the 5′-3′ degradation pathway order, we excluded the Ccr4–Not sensitive genes from the analysis and turned to examine transcription along the depletion time-courses of these factors.

In all three cases, we observed a reduction in transcription, but strikingly, the timing order of the observed reduction recapitulated the observed order in the case of total mRNA, namely—Not1 caused the most immediate decline in recently-transcribed mRNA, followed by Dcp2, and then by Xrn1 (*t_$\frac{1}{2}$_* of 33′, 52′ and 70′ respectively; Figure 5G). Having excluded an artifact due to different protein depletion kinetics (Supplementary Figure S5G), this result suggested that the difference in accumulated mRNA between the strains is due to the earlier onset of the adaptation response when Not1 is depleted compared to Dcp2 depletion and in Dcp2 relative to Xrn1 depletion.

While the reduction in transcription affects the entire genome, there was still significant transcript-to-transcript variation in the exact dynamics (Figure 5G). We reasoned that if the earlier reduction in transcription explains the reduced degree of mRNA accumulation in Not1 relative to Dcp2 and relative to Xrn1, this relationship should also hold per transcript, namely, genes whose transcription is decreased faster in Dcp2 compared to Xrn1 should accumulate less mRNA in Dcp2 compared to Xrn1. To test this hypothesis, we calculated the difference between cumulative changes in recently-transcribed mRNA during the accumulation phase (<60 minutes, ‘Δnew’), and the difference in maximal total mRNA during the accumulation and adaptation phase (<90 minutes, ‘Δmax mRNA’, Figure 5H). We then compared these measures between the different strain pairs and found a strong correlation (Figure 5H, *r* = 0.4, *P* < 10^–170^), i.e. genes with reduced transcription in Dcp2 depletion compared to Xrn1 depletion accumulated less mRNA, as expected. This was also the case (albeit to a lesser degree) when we examined the differences between Dcp2 and Not1 depletions (*r* = 0.31, *P* < 10^–98^, Supplementary Figure S5H).

Taken together, these results point to a global mRNA accumulation pattern in response to a perturbation along the 5′-3′ degradation pathway. The degree of mRNA accumulation can be explained by the timing of the transcription response, and furthermore, the onset time of the response is earlier when upstream factors in the 5′-3′ pathway are perturbed.

Finally, we looked for sets of genes whose transcription decreased faster or slower than average and found a clear signature of G1- and S-phase genes (Figure 5I) (84). For further results and analyses regarding the cell cycle, see supplementary notes and Supplementary Figures S6 and S7).

## DISCUSSION

We set out to study the mechanism of mRNA homeostasis that was observed in multiple conditions and organisms. The literature surrounding the question of feedback mechanisms between mRNA degradation and transcription is largely based on steady-state measurements. As a baseline, when we examined Xrn1 knockout we recapitulated a previous observation of a genome-wide reduction in degradation and transcription rates resulting in unchanged global mRNA (Figure 2). We reasoned that to study a feedback mechanism, steady-state measurements can be insufficient and potentially lead to incorrect interpretations. Therefore, we developed and applied a high-throughput sequencing-based implementation of the widely used cDTA technique (Figure 1). This allowed us to monitor total and recently-transcribed mRNA in detailed dynamic settings for hundreds of samples, resulting in the biggest resource of metabolically-labeled mRNA in yeast to date. Applying cDTA-seq to cells undergoing rapid Xrn1 depletion (Figure 3A), we observed pronounced mRNA accumulation in virtually all transcripts, followed by a return to normal wildtype levels. The labeled mRNA profile provided by cDTA-seq revealed a striking reduction in transcription roughly 60 minutes following Xrn1 depletion (Figure 4). Finally, when we applied cDTA-seq to cells in which different RNA processing factors were depleted, we found that the response to Xrn1 depletion is not unique; detailed dynamic measurements revealed that the depletion of decapping and deadenylation factors results in a similar initial increase in mRNA levels. However, while the initial response was similar, the reduction in transcription occurred earlier when upstream factors along the 5′-3′ degradation pathway were perturbed (Figure 5).

We focused on Xrn1 for two main reasons. First, it was implicated in mRNA homeostasis in two important but incongruent works by the Choder (19) and Cramer (18) groups. Secondly, Xrn1 degrades a large proportion of mRNA molecules in eukaryotic cells (6), so we expected a considerable response. Indeed, Xrn1 knockout exerts the most extreme alterations to mRNA profiles in published systemic knockout studies (Supplementary Figure S2A). Despite our technique being more akin to the cDTA protocol used by the Cramer group (Figure 1G), our data support the results from the Choder group, namely that mRNA levels are unchanged when cells lack Xrn1 and that degradation and transcription rates are significantly reduced (Figure 2).

To understand this homeostatic response, we applied cDTA-seq to cells undergoing rapid Xrn1 depletion (Figure 3A). We observed pronounced and transient mRNA accumulation immediately after the perturbation. To our knowledge, this is the first time that such a detailed view of a transient increase in mRNA levels is observed. Strikingly, in similar depletions of other factors (but not all, Figure 5E) cells revert to almost exactly the same mRNA levels they began with, suggesting some form of perfect adaptation taking place (85,86). Further dynamic data in different settings will be useful to model this possibility. Importantly, this response does not seem to be a normalization artifact as it was measured in multiple different experimental batches and conditions (Figures 3, 5, and Supplementary Figure S7), and it was observed by single-molecule FISH in four different transcripts (Figure 3). The observed reversion to normal levels was harder to verify by smFISH, as cells lacking Xrn1 accumulate deadenylated mRNA molecules (8,90) that also interact with the FISH probes. This makes the direct counting of polyA(+) molecules by FISH and microscopy challenging. As an alternative that circumvents direct molecule counting, we used polyA probes and FACS to measure the overall quantity of polyA RNA in cells and observed a temporal response consistent with the cDTA-seq signal (Supplementary Figure S3D).

Having excluded normalization, increased growth, or compensatory degradation as possible explanations for the reduction in mRNA levels, we could explain the return to wildtype levels by a global reduction in transcription roughly 60 min following Xrn1 depletion together with dilution by continued growth (Figure 4). The genome-wide nature of the reduction strongly supports a general mechanism for transcriptional regulation, rather than the existence of multiple gene-specific feedback loops. This result is further supported by a recent report that uses aneuploid cells to distinguish between these two possibilities (87).

Importantly, the observed delay in transcription reduction argues against the direct involvement of Xrn1 in this transcriptional reprogramming, as Xrn1 is degraded rapidly from cells (Figure 3A), but the transcriptional response occurs an hour later (Figure 4). Conversely, when we depleted Rat1 (the 5′-3′ exonuclease operating in the nucleus that is involved in transcription termination (88)) we observed an immediate reduction in transcription (Supplementary Figure S6B). Alternatively, Xrn1 was hypothesized to indirectly affect global transcription through post-transcriptional regulation of Nrg1 (18). Therefore, we repeated the Xrn1 depletion time course experiment in Δnrg1 and Δnrg2 strains but we did not observe any difference in the total mRNA dynamics or in transcription (not shown), arguing against Nrg1/2 involvement in the feedback.

To further dissect the response, we expanded our experiments to include the depletion of dozens of RNA-related factors (shown in Figure 5A, and others, not shown). This expansive view revealed various response dynamics (Figure 5D, E) and will hopefully prove useful to understand different phenomena related to mRNA processing. Focusing on the adaptation response we observed in the wake of Xrn1 depletion, it was clear that Dcp2 and Not1 depletion elicited similar cellular reactions (Figure 5F). The observation that Xrn1 depletion caused the most significant accumulation of mRNA amongst all the factors we depleted, and that this accumulation is similar to the response upon Dcp2/Not1 depletion, argues that the 5′-3′ pathway is the major pathway regulating mRNA levels in exponentially growing yeast in rich medium. Therefore, in our analysis, we put aside potential compensations by alternative degradation pathways (e.g. Not1-sensitive genes, Figure 5C, F), and focused on the 5′-3′ degradation pathway.

**Figure 6.**
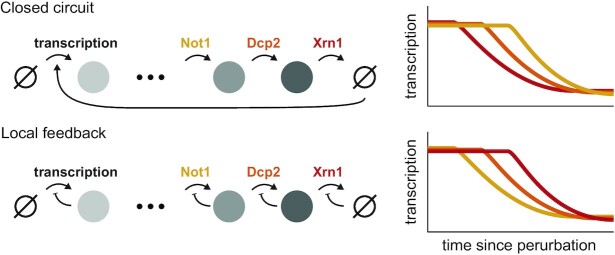
Dynamic measurements distinguish between different feedback models. Temporal offsets between different perturbations suggest a local rather than a closed-circuit feedback mechanism. Two toy models that result in global mRNA feedback are presented. In the closed-circuit model (top) transcription is coupled to degradation directly, while the local feedback model (bottom) assumes that each stage self-regulates. The steady-state behavior of both models will be similar, but dynamic measurements can be used to distinguish the two by the delay in the propagation of the interference back to transcription. Our data are more consistent with a local feedback model (see Figure 5).

Strikingly, when factors along the 5′-3′ mRNA degradation pathway were depleted, the mRNA response began essentially the same, but seemed to be attenuated in the order of the factor along the pathway (Not1 < Dcp2 < Xrn1). Consistent with the reduced accumulation of mRNA along the 5′-3′ pathway, we observed a delayed reduction in transcription, that also followed the order of the 5′-3′ degradation pathway (Figure 5G), parsimoniously explaining the reduced accumulation of mRNA (Not1 < Dcp2 < Xrn1). We interpret these results to suggest that cells do not monitor accumulated deadenylated and decapped mRNA, or downstream byproducts such as p-bodies (89), for if this was the case, transcription inhibition would ensue earlier in the Xrn1 depletion time course (Figure 6). Extending the logic of this argument, and neglecting alternative degradation pathways, the agreement between the 5′-3′ mRNA degradation pathway order and the onset time of the transcription adaptation response suggests that cells monitor a precursor that accumulates upstream of this pathway.

This model, when extrapolated, suggests local feedback along the lifecycle of the mRNA, rather than a closed-circuit feedback mechanism (Figure 6). Specifically, the model posits that accumulation of decapped mRNA inhibits decapping by Dcp2 and that subsequent accumulated capped mRNA inhibits deadenylation by Ccr4–Not, which will explain the timing offsets we observed. There are several interesting options upon consideration of this prediction: (i) 5-AMP released by Ccr4–Not deadenylation (90) may be important for proper cellular metabolism in general or DNA replication specifically. A rough calculation revealed that polyA-bound adenosine is within one order of magnitude of the amount of free ATP in cells (91). (ii) As previously suggested, polyA binding protein (Pab1) could be involved in the sensing mechanism (30,31). Notably, the Pab1 depletion data we presented here (Figure 6) argue against this hypothesis, but it requires further scrutiny, and we have not directly tested the nuclear analog - Nab2 (92). (iii) In a similar vein, an imbalance in mRNA nuclear export might be caused by polyA mRNA accumulation, which in turn causes an accumulation of nuclear mRNA that was recently suggested to directly affect transcriptional throughput (93,94). (iv) Ribosomes are essential for growth and proliferation, and free ribosomes were suggested to play an important role in this regulation (95,96). Cells may monitor free ribosomes which are presumably reduced as poly-A mRNA accumulates and binds them. (v) Alternatively, an imbalance between ribosomal proteins and rRNA can cause nuclear dysfunction and cell-cycle progression defects (97). Such an imbalance can arise from the over-production of ribosomal proteins due to mRNA accumulation. (vi) Last, the Ccr4–Not complex has been suggested to be a hub affecting transcription, translation, and degradation (33). A functional diversion of the Ccr4–Not complex itself due to accumulated mRNA may cause the transcriptional response.

Finally, upon examination of the dynamic response to interference in the 5′-3′ degradation pathway, we found a unique and transient signature of G1- and S-specific genes whose transcription reduced faster than average (Figure 5I, Supplementary Figure S6). When we prevented cells from iterating through the cell cycle only a modest decrease in transcription was observed following Xrn1 depletion (Supplementary Figure S7). Both lines of evidence are circumstantial, but together they point to a potential role for the cell cycle in the observed feedback. Cell cycle checkpoints are known to monitor cell size, nutrients, DNA damage, and proper chromosomal and cellular geometry (98), and it is possible that the accumulation of mRNA (or some other element) feeds back into one of these sensors or to a yet undescribed checkpoint. Furthermore, a cell-cycle coupled mechanism for regulating global transcription during the S phase was previously described (55,99). Since much of the molecular details of these checkpoints are known, testing the link between the transcriptional adaptation we observed in response to mRNA accumulation and the cell cycle is an interesting avenue for future research.

Taken together, our results suggest a model (Figure 6) in which input to the 5′-3′ mRNA degradation pathway in cells is monitored, and once a critical threshold is met an adaptive transcriptional response ensues, allowing cells to reestablish proper mRNA levels. More broadly, while mRNA homeostasis observations and functional experiments were mostly conducted in yeast, there is a growing body of literature that suggests this phenomenon is general (16,30,93,100). Notably, the factors we studied here along the 5′-3′ mRNA degradation pathway are highly conserved. A better understanding of the feedback mechanism employed by cells will likely have broader implications and might be relevant for critical processes in health and disease such as proliferation, apoptosis, and viral immune response (101). The data we presented here argue that detailed dynamic measurements are important for a deeper understanding of global mRNA regulation.

## DATA AVAILABILITY

Sequencing data are available under GEO accession number GSE196937. Scripts and summary data for generating all the figures and supplementary figures in this manuscript are available at 10.5281/zenodo.6528287. Raw microscopy images are available upon request.

## Supplementary Material

gkac411_Supplemental_Files
