# Supplementary material for: Transcription feedback dynamics in the wake of cytoplasmic mRNA degradation shutdown
Source: Nucleic Acids Res. 2022 May 30;50(10):5864–80. doi: 10.1093/nar/gkac411 (PMC9177992; doi:10.1093/nar/gkac411)
Supplement: gkac411_Supplemental_Files [file gkac411_supplemental_files.zip › xrn1_supplementary_material.pdf]

Transcription feedback dynamics in the wake of cytoplasmic mRNA degradation shutdown

Supplementary Material

Supplementary Figures **2**
Figure S1. cDTA-seq validation and analysis 2
Figure S2. Xrn1 knockouts analyses 5
Figure S3. Transient mRNA accumulation upon Xrn1 depletion 8
Figure S4. Exploring non-transcriptional explanations for the reduction in mRNA levels 10
Figure S5: The transcription adaptation response along the 5'-3' branch 12
Figure S6: Cell-cycle signature in the transcription adaptation response 14
Figure S7: Reduced transcription response in G1-arrested cells 16
Figure S8: Supplementary auxin depletion western blots 18

Supplementary Tables **19**
Table S1. Yeast strains used in this study 19
Legend / comments 20
Table S2. Oligonucleotides used in this study 21
Table S3. FISH probes 22
Table S4. Experimental details 22

Supplementary Notes **23**
Half-life estimation from 4tU labeling data 23
First-order model predictions when degradation is reduced but production is not 25
Not1 sensitive transcripts 26
Cell cycle signature analysis 26
Xrn1 depletion in a G1-arrested population 27

Supplementary References **29**

Supplementary Figures

Figure S1. cDTA-seq validation and analysis

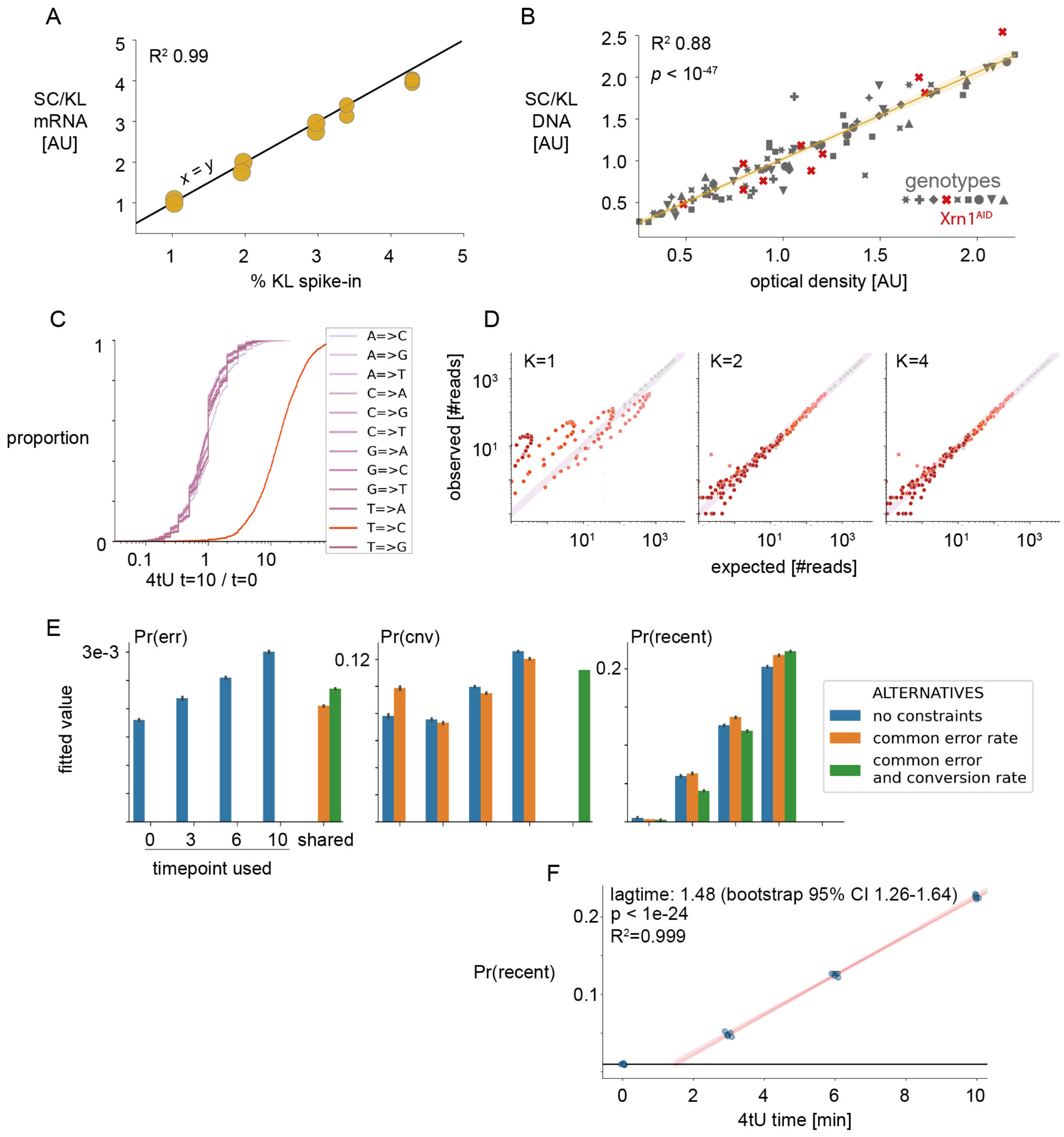

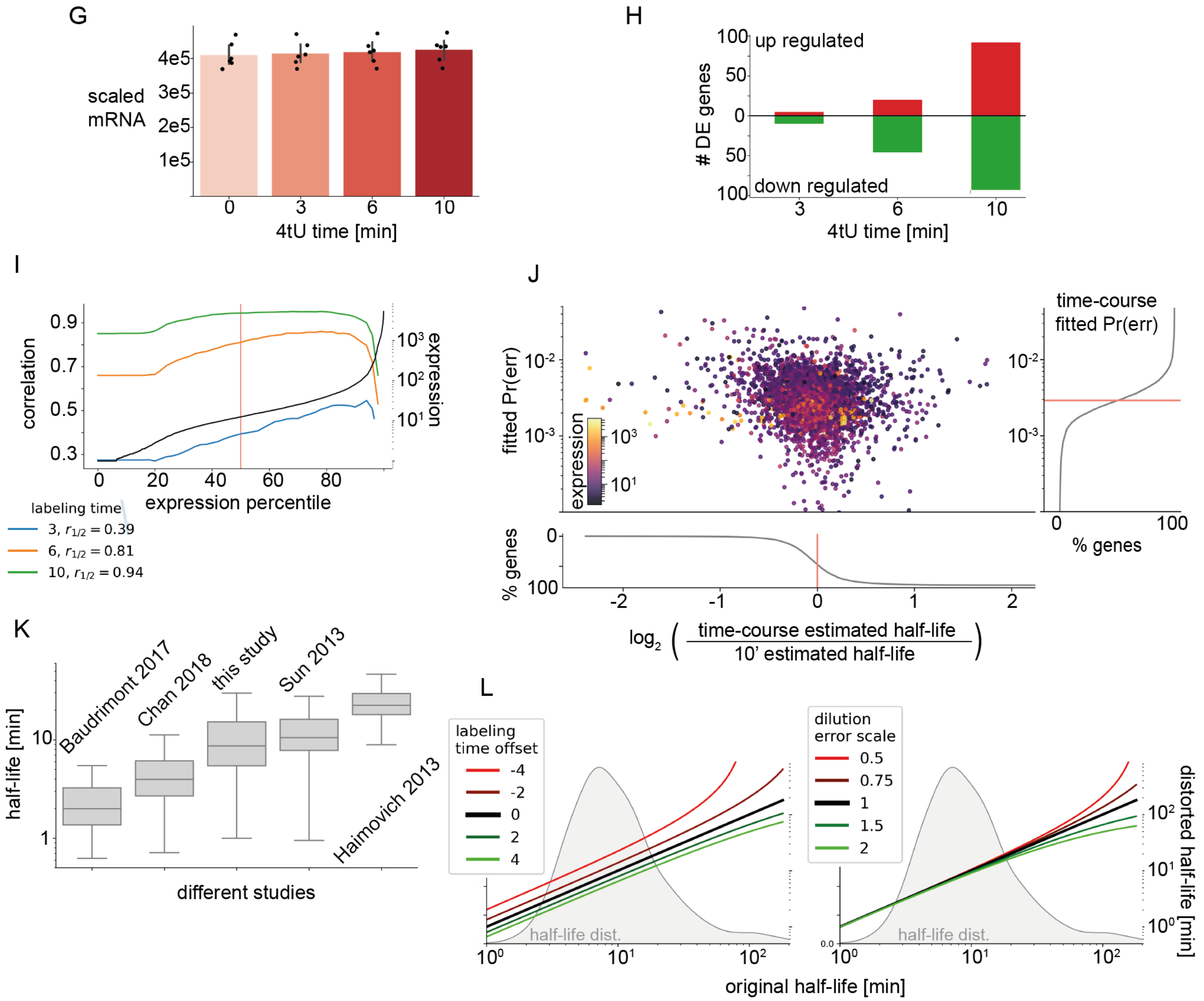

Supplementary Figure 1: cDTA-seq validation and analysis

A) Spike-in read ratio is linear. An SC sample was split and spiked in with varying amounts of KL cells (1-5%, x-axis). The ratio of mRNA reads from SC and KL (y-axis) is linear in this range (N=2, R^2=0.98, the black line is x=y).

B) OD is a good proxy for the number of cells. Each marker is one of 93 samples with various genotypes (markers) in various conditions (methods). Samples' optical density (OD, x-axis) was measured, they were spiked in with KL cells, DNA was extracted and the SC/KL DNA ratio (y-axis) is plotted against their measured OD (R^2=0.88, $p < 10^{-47}$, the yellow line is linear fit, shaded with 95% CI).

C) Only T→C observations are changed after 4tU labeling. Observed deviations from the reference were counted per transcript. The change in this count after 10 minutes of 4tU labeling is plotted as log fold change (x-axis) cumulative distribution across all transcripts (y-axis).

D) A 2-component BMM is sufficient to explain the conversion data. The binomial mixture model was fitted to the data (after 10' of labeling) with a varying number of mixture components (shown are 1, 2, and 4). Given a fitted model and the distribution of observed Ts per read, one can plot the expected number of reads per combination of Ts and converted bases (y-axis) vs. the actual number of observed reads per each combination (x-axis).

E) Alternative parameter sharing schemes show dominant change is due to p_r. 2-BMM was fitted to the 4tU time course data without any shared parameters (i.e. full model per sample, blue), with a shared error rate ("ε", orange), or with a shared error rate and a shared conversion rate ("ξ", green). In all cases, the Pr(recent) ("p_r") fitted parameter (right) correlates with 4tU labeling time (x-axis). N=6.

F) 4tU lag-time estimation. Fitting a linear model to the time-course data (confidence interval from data resampling) predicts that the labeling lag time is ~1.5 minutes.

G) No change to global mRNA. Spike-in scaled estimates of mRNA levels in samples along the 4tU time course show no changes to overall mRNA levels.

H) Differentially expressed gene increase with 4tU (<200 genes at 10'). DESeq2 differential expressed genes compared to t=0 along the 4tU time course with 6 replicates. After ~10 minutes of labeling, ~180 transcripts are up (red) or down (green) regulated compared to t=0. Enriched GO annotations in down-regulated genes: ribosome

(57/305, 4.3e-09), RNA binding (35/395, 4.4e-09), rRNA export from nucleus (10/18, 5e-09), rRNA binding (10/44, 7.5e-08), maturation of SSU-rRNA from tricistronic rRNA (9/69, 6.3e-05), and in up-regulated genes: cellular amino acid biosynthetic process (19/97, 4.3e-09), arginine biosynthetic process (6/11, 6.7e-07), glucose import (8/30, 1.2e-06), glucokinase activity (4/5, 3.2e-05), hexokinase activity (4/5, 3.2e-05).

I) Correlation between whole-time-course and single-sample-estimate is high between 6'-10' labeling. The half-life of each transcript was estimated with the full time course or with a single time point (3',6',10'), and the correlation between these estimates (y-axis) was plotted as a function of expression cutoff (x-axis). For example, at x=0.2, the correlation was performed only considering the top 80% of expressed transcripts. The black line denotes the expression levels [AU, log scale].

J) Pr(err) is not correlated to the difference between whole-time-course and single-sample-estimate, nor expression levels. Each transcript time course data was fitted with a model allowing for a transcript-specific error rate ("ε"), rather than a global one. Fitted values (y-axis, CDF with median in red on the right) range from 0.001 to 0.007 which are at least an order of magnitude lower than the fitted conversion rate ("ξ", ~0.11). Differences between transcripts are not correlated to half-life error estimations (x-axis, log fold change of half-life estimate based on full time course vs. half-life based on a single 10 min time point). Differences are also not correlated to expression levels (log scale, color-coded).

K) Global differences in half-life distributions between studies. The distributions of half-life estimates (in minutes, y-axis) are box-plotted for 5 different studies to highlight the different estimates scales.

L) Half-life estimation is robust to global parameter estimations. The theoretic half-life estimation (right y-axis) is compared to the actual half-life (x-axis), assuming different errors in global parameter estimations. For example, if cell growth is actually slower by a factor of two compared to the rate used when estimating half-life, the distortion is given by the bright green line on the right panel. Background smoothed histogram depicts the distribution of half-lives across the genome, guiding the eye to where most transcripts reside.

Figure S2. Xrn1 knockouts analyses

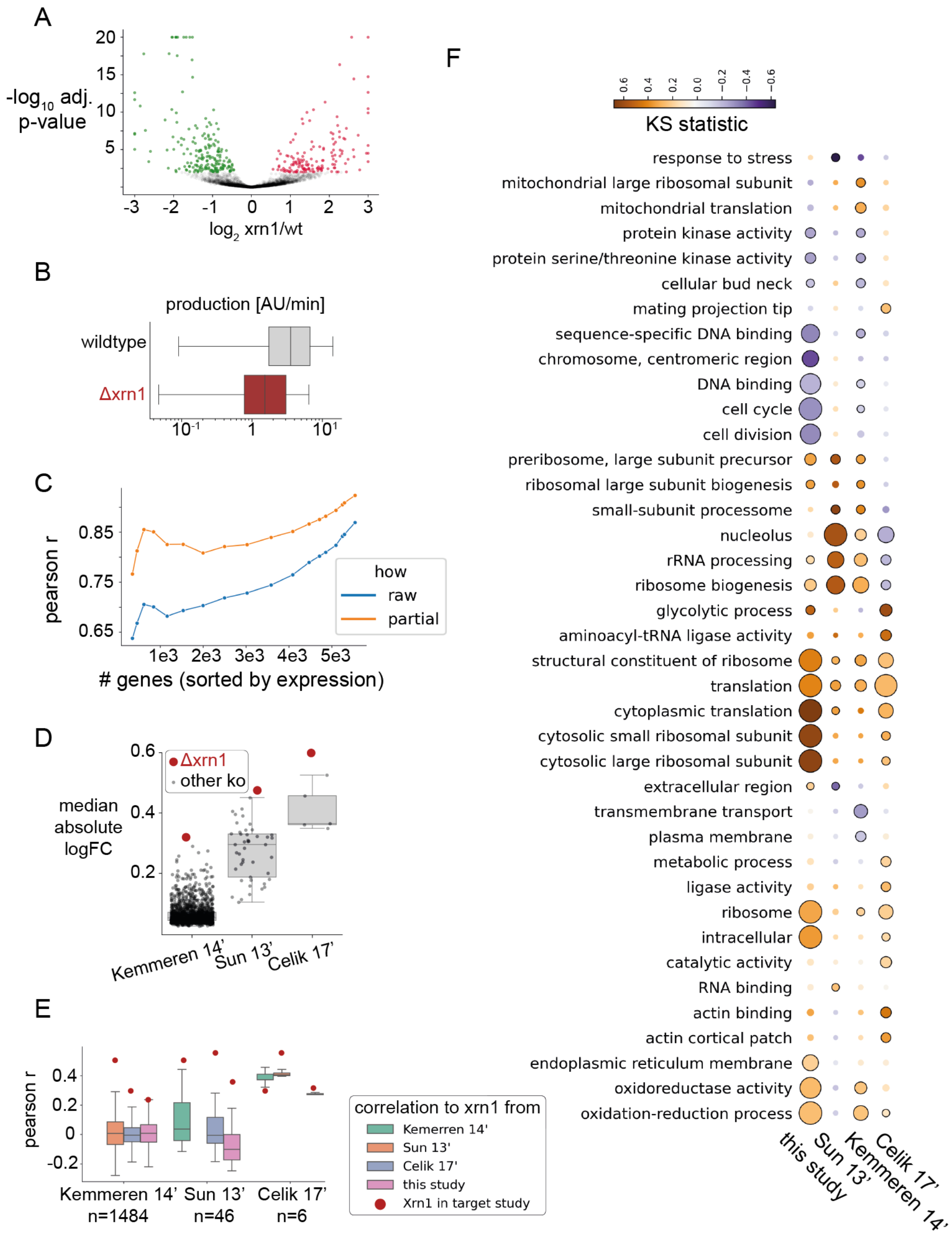

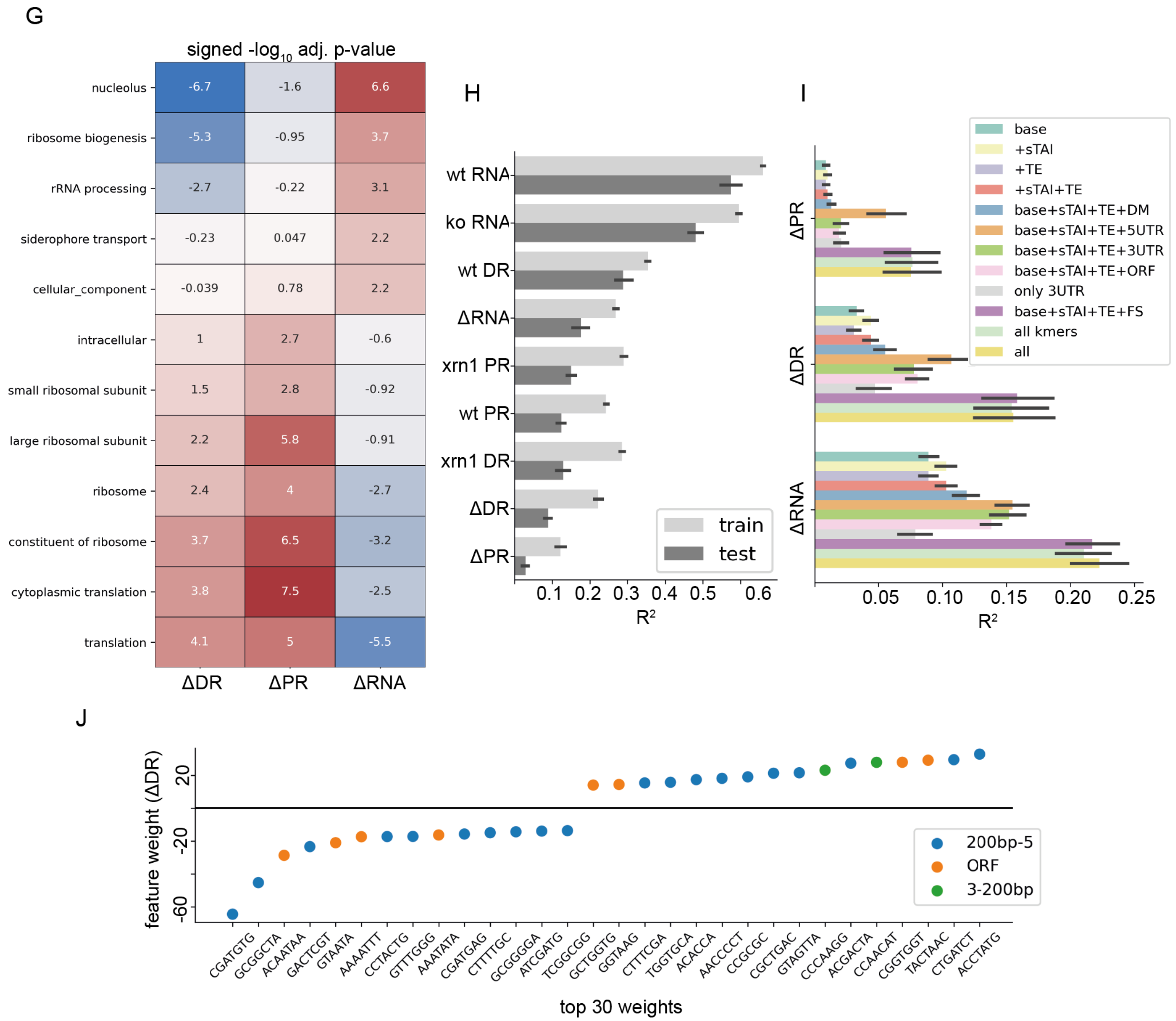

Supplementary Figure 2: Xrn1 knockouts analyses

A) Xrn1 knockout differential expression. Comparing two wildtype biological replicates and two biological replicates of two different clones without Xrn1, ~400 transcripts were significantly changed (adjusted $p < 10^{-2}$ (1)).
B) Inferred production rates are significantly reduced in Xrn1 knockout. Transcript production rate distributions, as inferred from mRNA levels and estimated degradation rates.
C) Correlation between change to production and degradation rate. Conditioned on genes with expression > threshold (n - number of genes above the threshold). The orange curve is the partial correlation (2), conditioned on mRNA levels.
D) An extreme transcriptomic signature for Xrn1 knockout. In three different knockout studies in yeast (x-axis), Xrn1 knockout (red dots) exerts the largest effect on the transcriptome as measured by the median over the absolute log2 fold change relative to wildtype strain (y-axis).
E) Correlations between studies. Correlating an Xrn1 KO from each "source" study (box color) to all KO strains in other studies ("target study", x-axis) generates a distribution of correlations (boxes). For example, correlating Xrn1 from Sun 13' with all KOs in Kemerren 14' generates the left-most orange box plot. Xrn1 in the target study is highlighted as a red dot, demonstrating that while the correlation between studies is low, Xrn1 is still the most significant correlation in most comparisons.
F) Enriched functional sets in knockout studies are incongruent. Enriched sets in up- or down-regulated genes in each study (top 15 per study). Circle size is proportional to negative log-, BH-adjusted- p-value (Kolmogorov-Smirnov test of set relative to overall distribution). Color denotes the signed KS statistic (orange - higher than average, purple lower than average).

G) Extreme gene sets in Xrn1 KO changes. All GO annotations (rows) were tested for having extreme distributions in their degradation rates, their mRNA levels, or their production rates (columns) using a Kolmogorov-Smirnov test. BH adjusted q-values are color-coded such that below-average changes are blue, and above-average changes are red (color bar). Numbers indicate log10 q-value. Only significant sets are shown (rows).
H) Explaining observed transcript response to Δxrn1. Trying to explain various observed RNA measurements (y-axis) with a sparse linear model (LASSO) using various transcript features and sequences (see supplementary note on modeling Δxrn1 changes). The X-axis is the % explained variance by the model prediction (R^2), on the training data (light gray, 90% of genes in 10 random samples), or the remaining test data (dark gray, 10% of genes).
I) Sequence features are sufficient for predicting xrn1 effects. Plotting model performance (x-axis, R^2), as in (H) using different subsets of features. K-mer counts from ORF, 5', and 3' areas are sufficient for model performance (see supplementary note on modeling Δxrn1 changes).
J) K-mer examples. K-mers (x-axis) are used by the model for explaining changes to degradation rates, ordered by their relative weight (y-axis), and colored by their class (ORF/5'/3'). Showing only the top 30 out of ~200 LASSO-selected k-mers in this case (degradation rate changes).

Figure S3. Transient mRNA accumulation upon Xrn1 depletion

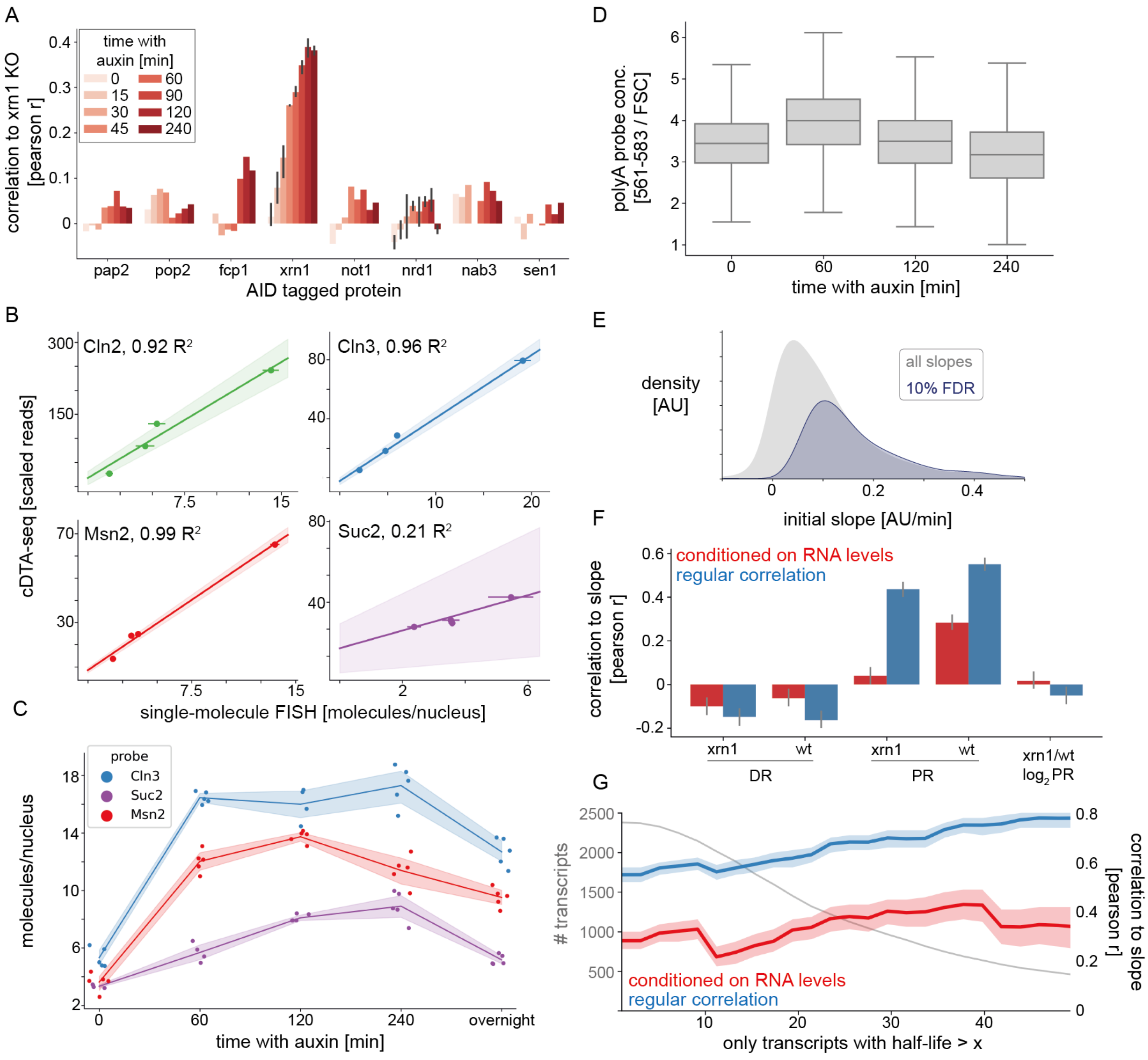

Supplementary Figure 3: transient mRNA accumulation upon Xrn1 depletion

A) Correlation of Xrn1 depletion to Xrn1 KO. log FC change from corresponding wt samples of multiple measurements following auxin addition of multiple degron strains were correlated to the Xrn1 knockout/wt log fold change. The correlation between the Xrn1-AID strain and the Xrn1 KO strain increases with time, i.e. these expression profiles become similar to that of the KO strain.
B) smFISH cDTA-seq comparison. smFISH measurements (x-axis) and cDTA-seq measurements (y-axis) are highly correlated. Points are one of four samples: Spt6-AID or Xrn1-AID with mock or auxin treatment for 60' minutes.
C) Slower RNA decrease in smFISH time course. Auxin time course (x-axis, last point is overnight) smFISH spots/nucleus (y-axis) for three probes shows the increase and slower decrease in total RNA.
D) polyA signal follows the cDTA-seq dynamics. polyA fluorescent probes quantified by FACS and normalized to forward scatter (y-axis)The distribution of 10k cells per time point shows an increase and decrease by 240 minutes, consistent with cDTA-seq data.
E) Slopes are positive and linear. The distribution of fitted initial slopes (x-axis, light grey) demonstrates that virtually all transcripts increase following Xrn1 depletion. We select transcripts that can be reasonably fitted with a linear fit (linear fit p-value, false discovery rate of 10%) for the following analysis. Colored dots indicate the slope (and FDR status) of the transcripts shown in (G).
F) Initial slope correlates to production rate, also after accounting for mRNA levels. Correlation (y-axis) between transcript slope to different transcript rates and measures (x-axis) was calculated with (red) or without (blue) conditioning on mRNA levels.
G) Initial slope correlation to production rate increases with transcript stability. As in (E), only examining subsets of transcripts with increasing stability (x-axis denotes the half-life threshold). The gray line indicates the number of transcripts (y-axis) used as the threshold increases.

Figure S4. Exploring non-transcriptional explanations for the reduction in mRNA levels

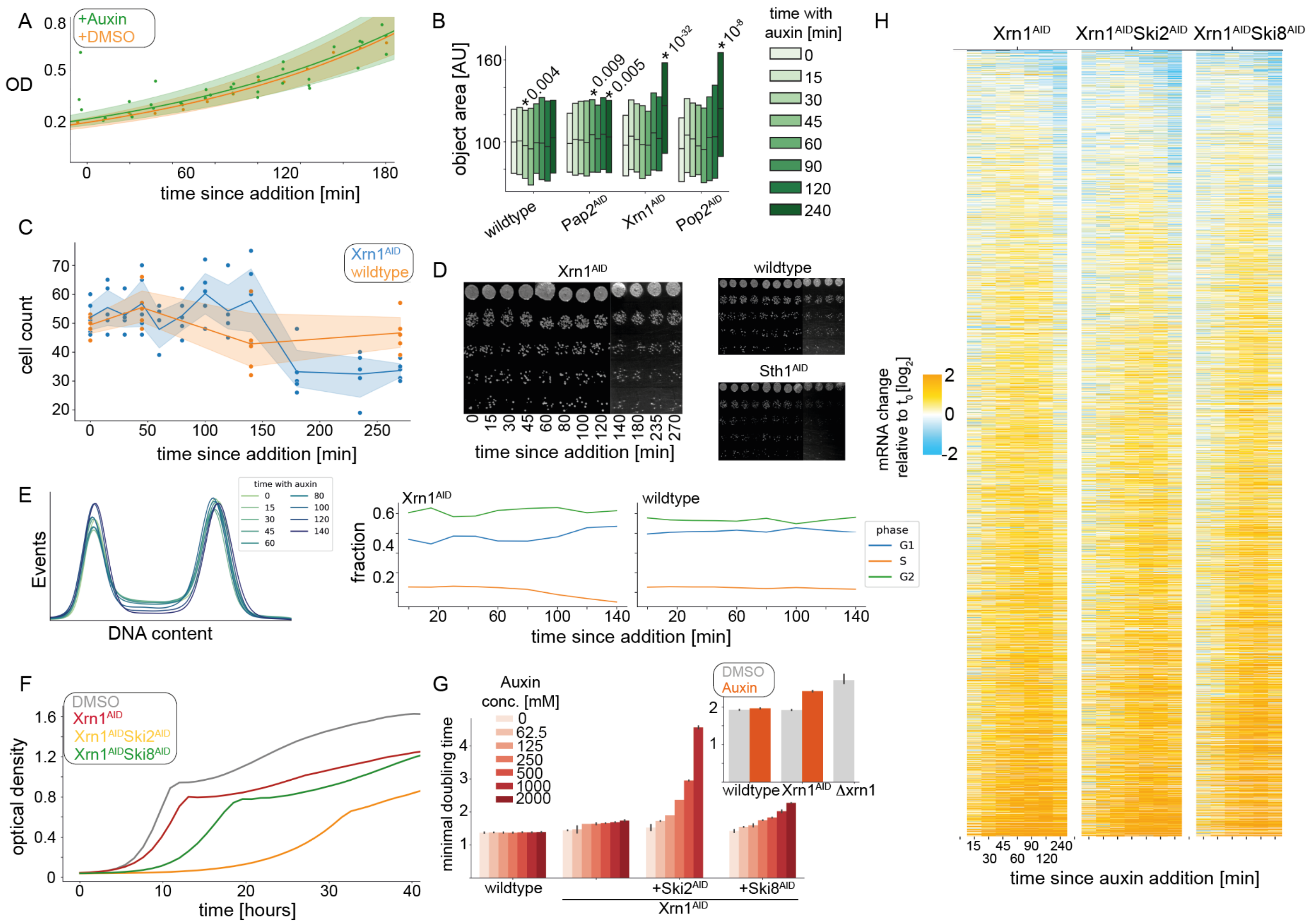

Supplementary Figure 4: Exploring non-transcriptional explanations for the reduction in mRNA levels

A) No immediate changes to OD or growth. OD measurements (y-axis) for samples grown on plates for 2 hours (x-axis starts at 120 minutes), and then supplemented with auxin (green) or not (orange). No significant changes in growth are observed within 3 hours.
B) Significant size changes were observed only after 4 hours. Several strains were imaged (supplementary methods), objects were segmented (3), and their area was quantified (y-axis). Each box group is a strain, colors indicate time since auxin addition. Each time course was normalized to the median area at time points 0, and 15. Significant t-test p-values are noted on the diagram. Xrn1 and Pop2 knockouts are known to have increased size (4–6).
C) No significant changes to cell counts within 2 hours. Cells were fixed in formaldehyde, vortexed, blinded, and manually counted on a hemocytometer (y-axis) along a time course with auxin (x-axis). A clear reduction in $Xrn1^{AID}$ cell counts is observed only after ~3 hours.
D) Colony-forming units show little change along an auxin time course. Cells were exposed to auxin for various durations (x-axis), serially diluted, and plated on YPD plates (no-auxin). A slight reduction is observed in the last three time points. As a control - no changes are observed in the wildtype strain, and a significant reduction is observed in an $Sth1^{AID}$ strain (as previously observed (7)).
E) No gross changes to cell cycle proportions in the first 2 hours following auxin addition. DNA staining (supplementary methods) of cells following auxin addition shows no gross changes to DNA distribution in an $Xrn1^{AID}$ population (left histograms). Fitted proportions of cells (supplementary methods) are fixed in a wildtype control (right) but potentially show a slight decrease in S-phase cells in $Xrn1^{AID}$ after ~80 minutes (left).
F) Xrn1-AID, Xrn1-Ski2-AID, and Xrn1-Ski8-AID growth curves. Growth curves (y-axis is OD, the x-axis is time in hours) for an isogenic strain (wildtype, grey), Xrn1-AID (red), double AID tag for Xrn1 and Ski8 (green), and double AID tag for Xrn1 and Ski2 (yellow) with final auxin concentration of 1000 μM.
G) Effects of different auxin concentrations on growth. Maximal doubling time (y-axis) recorded in the growth curves (examples in (D)) of the indicated strains (x-axis, same as in (D)) with different concentrations of auxin (colors). Inset: maximal doubling time (y-axis) for wildtype, Xrn1-AID, and Xrn1 knockout with (orange) and without (grey) auxin (2.2 mM).
H) No differences in RNA profile following auxin in double-tagged strains. RNA profile in time (x-axis) as log2 fold change relative to t=0 (color-coded) per transcript (rows) in the Xrn1-AID strain (left), and the double-tagged strains (+Ski2-AID, +Ski8-AID). Same as Figure 3D.

Figure S5: The transcription adaptation response along the 5'-3' branch

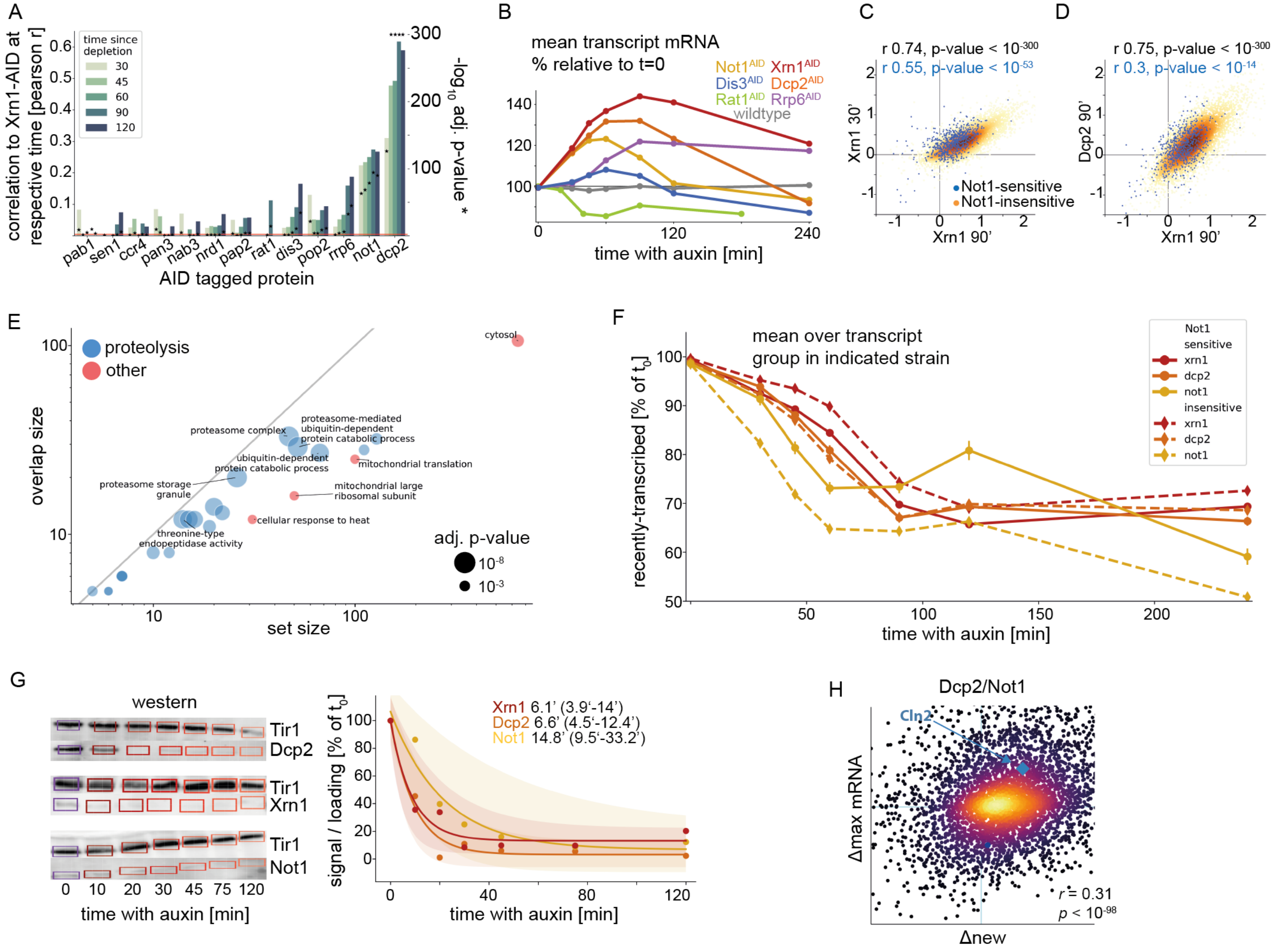

Supplementary Figure 6: The transcription adaptation response along the 5'-3' branch

A) Correlations to Xrn1. Correlations along the depletion time course (color legend) of various proteins (x-axis) to Xrn1 (in corresponding time points). The left y-axis denotes the Pearson correlation value(bars), the right y-axis denotes the -log10 p-value of the correlation (black stars). The red horizontal line is the adjusted threshold for significant p-values.
B) General mRNA response in time for selected factors. Average changes to transcripts' mRNA relative to t=0 (y-axis) along the time course (x-axis) for factors exhibiting significant correlation to Xrn1 (figure S5A). Same data as in Figure 5D, only including the 240' time point for completeness.
C-D) RNA log2 fold change between various samples. Scatters exemplifying correlations are shown in panel A and figure 6B. Same as Figure 6D. The set of Not1 sensitive transcripts (blue dots) is only exceptional in (D), but maintains an overall trend, suggesting an additive effect of 5'-3' interference response and Not1-specific response.
E) Not1-sensitive transcripts are enriched in proteolysis annotations. The set of Not1-sensitive genes was tested for significant overlaps with GO annotations (hypergeometric tests). Most enriched sets are proteolysis-related (blue). Log10 p-values are proportional to set marker size. The top 10 sets are named on the plot.
F) Transcription dynamics in Not1 sensitive transcripts. Same as Figure 6E (dashed lines here correspond to solid lines in Figure 6E), but also shows the dynamics of Not1-sensitive transcripts (solid lines). Due to the selection process, an opposite difference between Xrn1 and Not1 is generated (methods), but overall the trends observed throughout the recently-transcribed transcriptome are maintained also in this Not1-sensitive set.
G) Protein depletion kinetics do not explain the observed temporal cascade in transcription shutdown. Cells with AID-MYC-tagged proteins (Xrn1, Dcp2, Not1) were collected and subjected to a western blot against Myc. Tir1-Myc is used as a loading control. The ratio between target protein to the loading control is plotted as a function of time, normalized so initial levels are 100%. These data were fitted with exponential decay, and the decay rate estimate is shown in the legend, with 95% confidence intervals.
H) Recently-transcribed mRNA differences explain total mRNA differences between strains. Same as Figure 5H, but comparing Not1 to Dcp2.

Figure S6: Cell-cycle signature in the transcription adaptation response

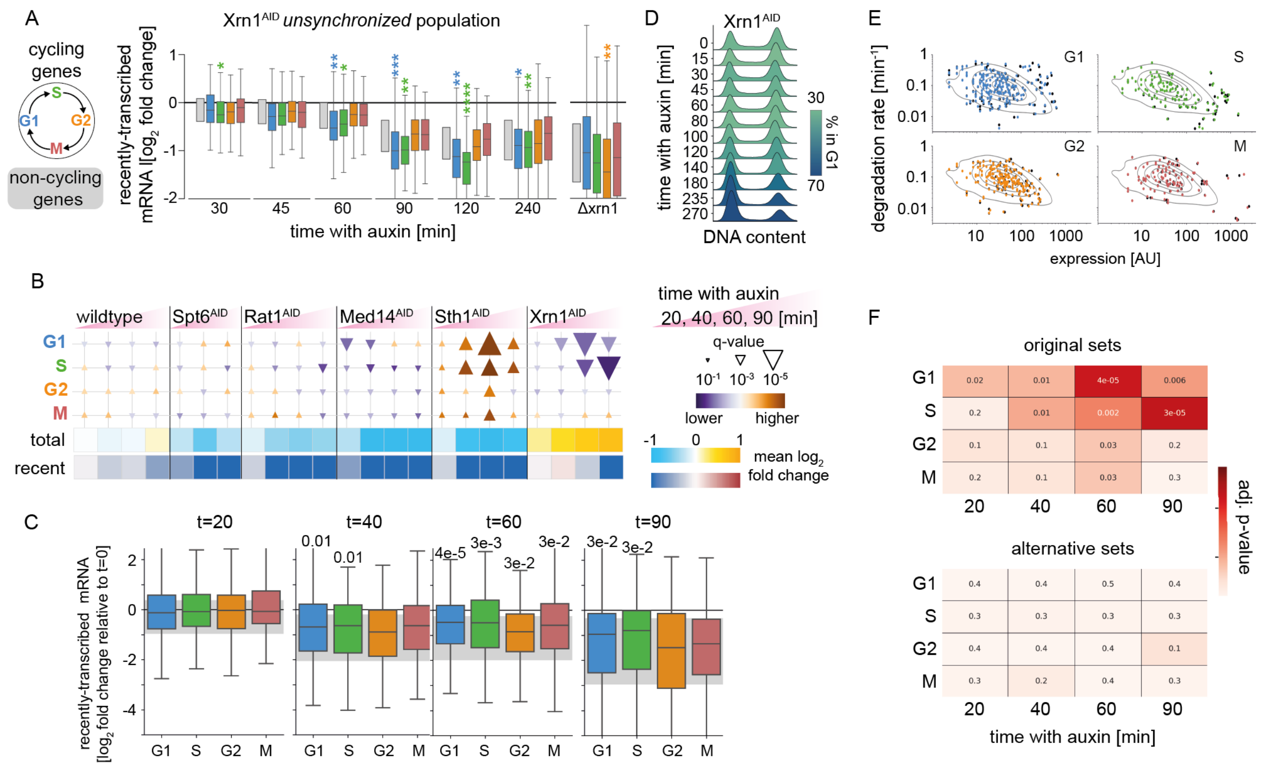

Supplementary Figure 6: Cell cycle signature in the transcription adaptation response

A) Cell-cycle signature following Xrn1 depletion in an unsynchronized population. Using data from the experiment shown in 4, we compare the distributions of changes in recently-transcribed mRNA (relative to t=0, i.e. no auxin, x-axis) of each set of cycling genes (colors, legend)(8) to the distribution of non-cycling transcripts (gray box is IQR). Different time points in this dataset are organized from left to right (x-axis labels). Significant deviations (Kolmogorov-Smirnov q-values) are marked with 1/2/3/4 colored stars if their q-value is smaller than 10^{-2}, 10^{-5}, 10^{-10}, and 10^{-15} respectively. On the right - the same analysis was applied to the Δxrn1 data shown in Figure 2.

B) The cell cycle signature is unique to Xrn1 depletion. We repeated the time course experiment on multiple different AID-tagged proteins (similar analysis as in fig 5I). Differences in log fold change to recently-transcribed mRNA between cycling gene sets (rows) and non-cycling genes denoted as colored triangles (purple/down - lower than non-cycling genes, orange/up - higher than non-cycling genes, size proportional to Kolmogorov-Smirnov q-value). Each triangle denotes the difference along a specific depletion time point (columns, x-axis time since auxin addition, same as in (A-C)). The bottom panels denote the average log fold change to mRNA and recently-transcribed mRNA in the same samples.

C) Sth1 shows a reversed G1/S signature. Repeating the analysis and visualization from (A) on the Sth1 depletion time course presented in (B) shows that the G1/S genes are higher than the non-cycling genes (IQR shown as a shaded gray background). Significant adjusted p-values are written above the respective box. The time point is indicated above each figure.

D) FACS analysis of DNA-stained unsynchronized Xrn1^{AID} cells exposed to auxin. DNA staining does not show any gross cell-cycle differences in the first 140 minutes. Only after 3 hours a shift to G1 is observed. Events histograms (y-axis) per 525nm filtered signal (x-axis, DNA was stained with SYBR green) are ordered along the time course following auxin addition (top to bottom) and are colored according to the percent of events associated with G1 phase DNA content (methods).

E) Selection of matched genes to cell cycle sets. The nearest-neighbor procedure was used to get a very close distribution of genes in the expression (x-axis) vs. degradation rate (y-axis) space. Colored dots denote the named cell-cycle set, black dots are the matched set from non-cycling genes.

F) Matched sets demonstrate that cell-cycle signature is not due to degradation rate or mRNA levels. Original sets (top), or matched sets (bottom) were subjected to the same analysis as in (B/Xrn1) - no significant differences were identified between matched sets and all other genes.

Figure S7: Reduced transcription response in G1-arrested cells

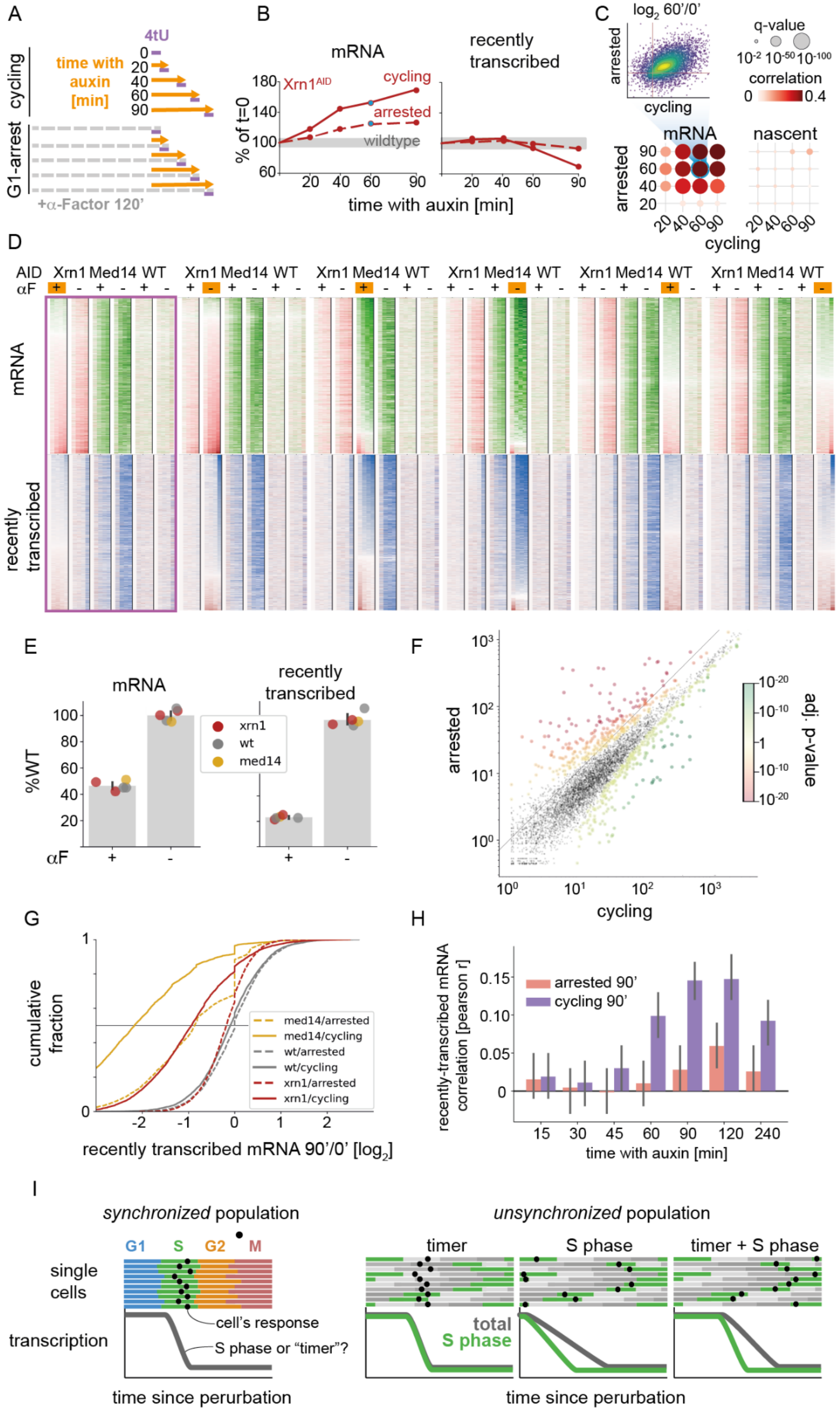

Supplementary Figure 7: Reduced transcription response in G1-arrested cells

A) Cell-cycle arrest AID/cDTA-seq experimental scheme. Cells were grown to mid-log phase and split into two cultures - with or without **α**-factor (a pheromone that arrests cells in G1). Cultures were then further split and auxin was added at indicated time points. All samples were subjected to a short 4tU pulse simultaneously and harvested for cDTA-seq.

B) Global changes to total and recently-transcribed mRNA following Xrn1 depletion in cycling and arrested cells. Average change relative to t=0 (y-axis) as a function of time (since auxin addition, x-axis), in cycling (solid line) and arrested (dashed) cells. Changes to total mRNA on the left (60' comparison shown in (C)), and changes to recently-transcribed mRNA on the right. The gray rectangle denotes the maximal deviation observed in the corresponding average (+SEM) calculated in the wildtype strain.

C) Correlations between cycling and arrested RNA profiles following Xrn1 depletion. Dot plots denote correlations between different time points along the two-time course experiments (x-axis: cycling, y-axis: arrested). Color proportional to Pearson *r;* dot size proportional to q-value (legend on top-right). A comparison of the 60' time point is shown as a scatter plot; each dot is a different transcript (colored by local density). Axes are the $\log_2$ fold change relative to t=0, which ranges between a 2-fold reduction and a 4-fold increase on both axes. Horizontal and vertical lines in the background denote no change (Pearson $r = 0.42$, $q < 10^{-300}$).

D) Cell cycle experiment data sorted by every sample. Each heatmap entry is the change in total mRNA (top, red/green) or in recently-transcribed mRNA (bottom, red/blue) relative to t=0. Rows are transcripts, columns are sorted by strains and then by time since auxin addition (20/40/60/90 minutes). Each heatmap is sorted by the signed maximal change in one of the samples (indicated in orange). For example, the top left heatmap (purple box) is sorted by the mRNA change in the Xrn-AID strain when it was arrested (+αFactor, orange).

E) Basal state of arrested cells. Total and recently-transcribed mRNA is significantly lowered in the arrested cells (+αFactor) compared to cycling cells (-αFactor), before auxin addition. Colors indicate different AID strains. Bars indicate the average value and error bars indicate the SEM (N=5). Mean decreases to 46.5% of cycling cells total mRNA (t-test $p < 10^{-7}$), and 23.1 % of recently-transcribed mRNA (t-test $p < 10^{-8}$).

F) Differential expression highlights the expected cell-cycle arrest signatures in mRNA profiles. Average expression per transcript (dots) of all strains and repeats in cycling (x-axis) or arrested (y-axis) cells. Data were analyzed with DESeq2 (1), and the adjusted p-values were used to color genes that are differentially expressed (red - significant in arrested, green - significant in cycling). GO annotations enriched in the down-regulated genes include: S: 2e-09, G2: 2e-08, ribosome biogenesis: 3e-08, cell wall: 8e-08, rRNA processing: 4e-07. GO annotations enriched in the up-regulated genes include: extracellular region: 2e-06, karyogamy involved in conjugation: 4e-06, fungal-type cell wall: 3e-05, cell wall organization: 0.0004, mating projection tip: 0.0005.

G) It is possible to measure a significant reduction in recently-transcribed mRNA in arrested cells. Cumulative distribution functions (CDFs) for change in recently-transcribed mRNA of the three strains (colored as in (A) and (B)) after 90 minutes of auxin. Each strain has a cycling sample (solid line) and an arrested sample (dashed line). As in Figure 5F, since the cycling wildtype 90' sample had too few reads, the 60' minute sample was used in this plot. The plot shows a significant decrease in recently-transcribed mRNA upon Med14 depletion from arrested cells (yellow dashed line).

H) Arrested cells' response does not look like a slower version of the transcription adaptation response. Changes in recently-transcribed mRNA following auxin addition to the Xrn1 strain were correlated to the changes in recently-transcribed mRNA in arrested/cycling cells from a different Xrn1 depletion time course (the one shown in figure (4)). While the correlation to the cycling cells is evident and significant (purple, Pearson p for 45', 60', 90', 120', 240' < 0.06, 10^{-9}, 10^{-20}, 10^{-21}, 10^{-9} respectively), in the case of the arrested cells, only the 120' is significant (pink, $p < 0.0002$).

I) Cell cycle data suggests a cell-cycle coupled timer sensing mechanism. In a synchronized population (left) a delayed transcriptional response can be explained by an apparent "timer" triggered by a cellular element accumulating since Xrn1 depletion, or by a specific cell cycle phase (S-phase as an example). However, dynamic measurements from an unsynchronized population can be used to distinguish these models by the differences between genes expressed along the cell cycle (in this case in S-phase, green) to non-cycling genes (dark grey). Our data suggest a model involving the cell cycle (G1/S) and a "timer" mechanism.

Figure S8: Supplementary auxin depletion western blots

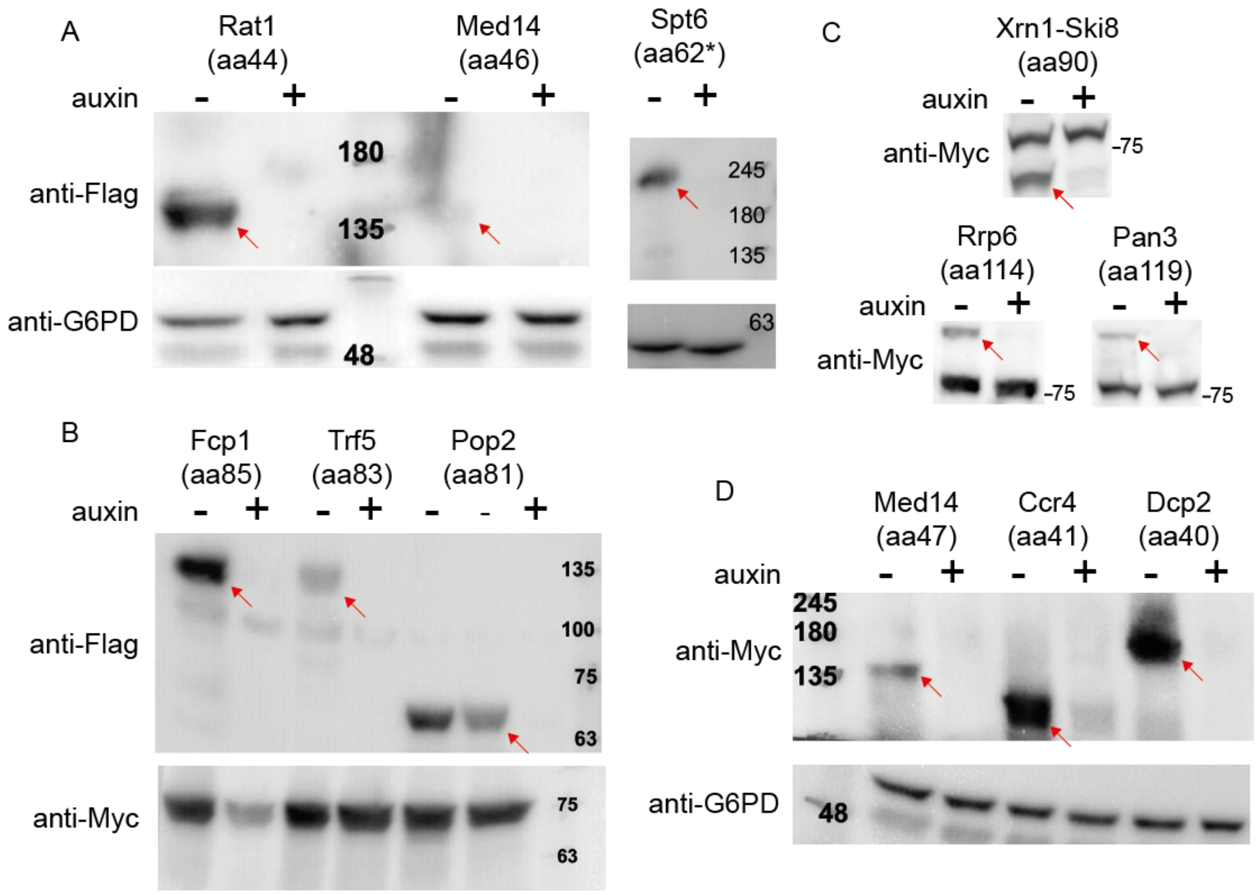

Supplementary Figure 8: Supplementary auxin depletion western blots

See specific comments in the strain table (table S1).

A) Western blot for Rat1, Med14, Spt6. Auxin was added to exponentially growing cultures for 1 hour. Samples were lysed and proteins were extracted for PAGE and western blot. Proteins are tagged with FLAG epitopes. G6PD is a loading control. Sizes in kDa are denoted on images. Red arrows denote the target band at the expected size.

B) Western blot for Fcp1, Trf5, Pop2. Same as in (A). osTir1-Myc was blotted as a loading control. Pop2 was loaded at x1 and x½ amounts without auxin.

C) Western blot for Ski8, Rrp6, Pan3. Same as in (B). Targets are tagged with a Myc epitope, osTir1 is used as a loading control (red star).

D) Western blot for Med14, Ccr4, Dcp2. Same as in (A). Targets are tagged with a Myc epitope, G6PD was stained as a loading control.

Supplementary Tables

Table S1. Yeast strains used in this study

All strains were derived from a gift from the Ulrich lab ("nf164") with the following genotype (DF, mat-a): his3-Δ200, leu2-3,2-112, lys2-801, trp1-1(am), URA3::TIR-9Myc.

NF number	Name	derived from	Genotype	Auxin growth rate	Western validation	RNA changes
nf227	XRN1-IAA*-MYC	nf164	XRN1-44AID9Myc::natNT	0.78, fig. S4	fig. 3	V
nf198	STH1-IAA*-FLAG	nf164	STH1-44AID9Flag::hphNT	0	ref (7)	V
nf189	SPT6-IAA*-FLAG	nf164	SPT6-44AID9Flag::hphNT	0	fig. S8[1]	V
aa40	dcp2-degM	nf164	DCP2-44AID9Myc::natNT	0.65	fig S5	V
aa44	rat1-degF	nf164	RAT1-44AID9Flag::hphNT	0	fig. S8	V
aa46	med14-degF	nf164	MED14-44AID9Flag::hphNT	0	fig. S8[2]	V
aa47	med14-degM	nf164	MED14-44AID9Myc::natNT	0	fig. S8	V
aa82	Pop2-AID	nf164	POP2-44AID9Myc::natNT	0.75	fig. S8[3]	V
aa85	fcp1-AID	nf164	FCP1-44AID9Flag::hphNT	0.45	fig. S8	V
aa72	NatΔXrn1	nf164	Xrn1::natNT	0.7, fig. S4	N/A	V
aa73	HygΔXrn1	nf164	Xrn1::hphNT	0.7, fig. S4	N/A	V
aa106	Xrn1-Flag-HygMX	nf164	Xrn1-44AID9Flag::hphNT	reduced		V
aa88	Xrn1-Ski2-AID	nf227	XRN1-44AID9Myc::natNT, SKI2-44AID9Myc::kanMX	0.1, fig S4		
aa90	Xrn1-Ski8-AID	nf227	XRN1-44AID9Myc::natNT, SKI8-44AID9Myc::kanMX	0.6, fig S4	fig. S8	
aa92	Nrd1-Flag-HygMX	nf164	NRD1-44AID9Flag::hphNT	0.44		V
aa93	Nrd1-Myc-NatMX	nf164	NRD1-44AID9Myc::natNT	0.44	fig. S8[?]	V
aa98	Sen1-Flag-HygMX	nf164	SEN1-44AID9Flag::hphNT	0.85		V
aa97	Nab3-Myc-NatMX	nf164	NAB3-44AID9Myc::natNT	reduced		V
aa113	Dis3-Myc-NatMX	nf164	DIS3-44AID9Myc::natNT	reduced		V
aa114	Rrp6-Myc-NatMX	nf164	RRP6-44AID9Myc::natNT	reduced	fig. S8	V
aa118	Ccr4-Myc-NatMX	nf164	CCR4-44AID9Myc::natNT	reduced	fig. S8[4]	V
aa91	Not1-Myc-NatMX	nf164	NOT1-44AID9Myc::natNT	0	fig. S5	V
aa116	Pab1-Myc-NatMX	nf164	PAB1-44AID9Myc::natNT	0		V
aa119	Pan3-Myc-NatMX	nf164	PAN3-44AID9Myc::natNT	reduced	fig. S8	V

Legend / comments

Auxin growth rate - is given as a fraction of the growth rate of the same strain without auxin (0 means no growth). “reduced” means that reduced growth was significant and observed but not accurately measured. Where a figure is noted then the data is available there for inspection.

Western validation - auxin depletion of indicated strains was validated by western. Summary images are found at indicated figures. Empty cells indicate that the western blot failed. Specific comments:

1. Spt6 western blot was done on a strain derived from this strain
2. Only a faint band was observed in strain aa46 (Med14), but it was at the right size, and was depleted when cells were exposed to auxin.
3. Western blot is of a different strain (aa81), indicating that C’-tagging of Pop2 with AID is effective.
4. Western blot is of a different strain (aa41), indicating that C’-tagging of Ccr4 with AID is effective.

RNA changes - a “V” denotes significant transcriptome changes when comparing auxin +/- samples (compared to a wildtype strain). In cases where transcriptional phenotype was mild (ccr4, pop2, NNS complex) - correlations between strains of the same complex provided evidence that the transcriptional changes are due to designated target depletion.

Table S2. Oligonucleotides used in this study

The following primer pairs were used to AID-tag the various strain shown in table S1:

Name	Sequence
xrn1-deg-F	CAATGCTGCTGACCGTGATAATAAAAAAGACGAATCTACTcgtacgctgcaggtcgac
xrn1-deg-R	TAAAGTAACCTCGAATATACTTCGTTTTTAGTCGTATGTTatcgatgaattcgagctcg
STH1-deg-F	AAATGAGTTTACTGATGAATGGTTCAAGGAACACTCTTCGcgtacgctgcaggtcgac
STH1-deg-R	ATATAGTCGTAAAAAAAAAAAACATGTGGTGATGAAAACGatcgatgaattcgagctcg
SPT6-deg-F	AAAATCTAACAGTAGTAAGAATAGAATGAACAACTACCGTcgtacgctgcaggtcgac
SPT6-deg-R	ATAATAAAATTAATAATAACAATGGACACTACATACGCATatcgatgaattcgagctcg
dcp2-f-deg	TTCAGGGTCTAATGAATTATTAAGCATTTTGCATAGGAAGcgtacgctgcaggtcgac
dcp2-r-deg	CATTTACAGTGTGTCTATAAAACGTATAACACTTATTCTTatcgatgaattcgagctcg
xrn1_ko_F	ACTTGTAACAACAGCAGCAACAAATATATATCAGTACGGTcgtacgctgcaggtcgac
rat1-f-deg	CAAGCAAAGTCGGTATGACAATTCAAGAGCAAATAGGCGTcgtacgctgcaggtcgac
rat1-r-deg	AACCTAAATTTACCATAAAATAAAATGCGCACGAGTAGTTatcgatgaattcgagctcg
med14-f-deg	CCATAATATCCTCAAAGTGGACTCGAACTCAAGTTCATCTcgtacgctgcaggtcgac
med14-r-deg	TCTCCTAAGGGATAGTAGCGCCGGTGACATTTTATTCGCTatcgatgaattcgagctcg
pop2-f-deg	CAAGTACCAAGGTGTCATATACGGTATTGATGGGGACCAAcgtacgctgcaggtcgac
pop2-r-deg	TTTTTTTTTAAAATTGTGTATACATATAGTACATAAATGAcgatgaattcgagctcg
fcp1-f-deg	TTCGCAGTTGGAGGAAGAGTTGATGGATATGCTGGATGATcgtacgctgcaggtcgac
fcp1-r-deg	CAATGAGGAAAATGTGTGGAAAGATACGGCATCTGAGCTGcgatgaattcgagctcg
Pap2-AID-fwd	CGAAGATGATGATGAAGATGGATATAATCCTTATACCCTTCGTACGCTGCAGgtcgac
Pap2-AID-rev	ATGTACAGTTCAGTGCATCATTTAAACAAAAAGGCACATAATCGATGAATTCGAGCTCG
Nrd1-AID-fwd	GAATATGCTTAACCAACAGCAGCAGCAACAACAACAAAGCCGTACGCTGCAGgtcgac
Nrd1-AID-rev	TTTTATGTACTATGAGCAAATAAAGGGTGGAGTAAAGATCATCGATGAATTCGAGCTCG
Sen1-AID-fwd	ATCTAGCCCATTTATCCCAAAAAAAAGAAAGCCTAGATCACGTACGCTGCAGgtcga
Sen1-AID-rev	TATATATGCAGGTATAATTCCTAACACTTTTACTTCAAGAATCGATGAATTCGAGCTCG
Nab3-AID-fwd	TGTTCAAAGTCTATTAGATAGTTTAGCAAAACTACAAAAACGTACGCTGCAGgtcgac
Nab3-AID-rev	TATAATGTACAAGAAATGGAAAAGATTGAAAAAAGGGAGTATCGATGAATTCGAGCTCG
ccr4-f-deg	ATTTGAATTTATGAAGACAAACACAGGCAGTAAGAAAGTAcgtacgctgcaggtcgac
ccr4-r-deg	GTACAGAGAGGAGGGAGGGAGTGGGATGAAAGTGTGCGGTatcgatgaattcgagctcg
not1_deg_f	CACCATCAATAGAAGGCAAACCCCTCTACAATCCAACGCAcgtacgctgcaggtcgac
not1_deg_r	CTGAAATCATGATTTTCGTATATAAATAAATGCAGTTTTTatcgatgaattcgagctcg

Table S3. FISH probes

Provided as an external excel spreadsheet.

Table S4. Experimental details

experiment	code	date	by	#samples	4tU [min]	auxin [mM]	Main figures	Supp figures
4tU time course	4tu-tc	30/07/2018	Daphna	24	0-10	N/A	1C-G	S1C-L
thiolutin test	thio-tc	19/7/2018	Daphna	4	6	N/A	1C	
klac calibration	klac-spikein	01/08/2018	Daphna	10	5	N/A		S1A
OD DNA calibration	dna-lib	N/A	Daphna	96	N/A	N/A		S1B
smFISH	fish1	15/08/2018	Alon	8	N/A	2.5		S3B-C
Xrn1 KO	xrn1ko	29/10/2018	Alon	6	9	1	2A-D	S2A-J,S5E
Screen1	exp13	14/04/2019	Alon	92	8.5	1	3(B-C,F-G),4B-D, 5B-C	S3A,S3E-G, S4H
Screen2	exp19_og2tc	12/10/2019	Omer	96	9.2	2.5	5B-I	S5
cell-cycle	cellcycle8	03/12/2018	Alon	80	9.3	2.5		S6B,S6C, S7
Xrn1 repeat	exp16-hu	25/06/2019	Alon	24	9	1	3B	

Supplementary Notes

Half-life estimation from 4tU labeling data

The following section describes the process by which we verified and tested various aspects of the half-life estimation procedures (Figures 1-2).

First, we fitted binomial mixture models with a varying number of components and found that two components (i.e. for old/new molecules) were sufficient to describe the observed distribution of T→C conversions (Figure S1D). We This model admits 3 parameters:

- The recently-transcribed fraction - p_r
- The background (sequencing) T→C conversion rate - ε
- The 4tU-induced T→C conversion rate ξ

Different samples along a 4tU labeling time course will obviously differ by their recently-transcribed fraction, but we wanted to verify that this is the only major difference between samples, i.e. that the error and conversion rate is common within an experimental batch and needs to be fitted once. We, therefore, fitted the data from the 4tU labeling time course allowing for each sample to have an individual set of parameters or requiring samples to share the error rate and the error+conversion rates (Figure S1E). In all cases, the recently-transcribed fraction was the single fitted parameter that varied the most, suggesting that the other parameters are largely time-independent. Furthermore, the constraint that samples will share the error and conversion rates caused only minor differences between the estimated recently-transcribed fraction, which is the critical parameter. This convinced us that these parameters (error and conversion rates) can be fitted globally once per experiment.

Next, when we fit the data with a 2-BMM model, we observe a linear increase in the fitted recently-transcribed fraction (p_r, Figure S1F), albeit with a time lag of ~1.5 minutes. Given that the recently-transcribed fraction should accumulate linearly for short labeling periods (assuming the model described in the methods section in the main text), we interpret this result to mean that there is a lag between the addition of the 4tU to samples and the measurements of recently-transcribed molecules. This could be due to an actual delay in the time it takes 4tU to sufficiently accumulate in nuclei, or due to polyadenylation and maturation time of the first labeled molecules. In any event, we use the time course data and fitted delay in subsequent experiments as a baseline assumption, adjusting the degradation rate equation from the main methods to include a time offset (t_0):

$$\delta = -\frac{1}{t-t_0}\ln(1 - p_r) - \gamma \quad (1)$$

Having verified that the cells are not disturbed from steady-state growth within several minutes of 4tU labeling (Figures S1G-H), we fit the half-life of individual transcripts using the 4tU time course data. We

perform a maximum likelihood estimation, iterating between fitting the global parameters (dilution rate, labeling lag time, and conversion rate) and transcript-specific parameters (half-life and error rate, which in this case we allowed to be transcript-specific). When this process converges to the maximum, we calculate a confidence interval for each half-life estimation assuming a quadratic log-likelihood around the maximum, or a linear log-likelihood if the maximum was constrained (i.e. too stable/volatile). These estimates are the ones shown in Figure 1F.

These half-life estimates were obtained using a 4-point time course in 6 replicates, but we want to estimate the half-life from a single measurement. We calculated the estimated half-life from individual measurements (i.e. no time course data), by fitting the recently-transcribed fraction per transcript using the 2-BMM model, and using equation 1 (assuming a constant error rate for all transcripts).

We found that a single measurement after 6'-10' minutes of 4tU labeling results in good agreement with the full time course estimate (Pearson r 0.66, 0.85 for 6', 10' respectively, Figure S1I). As expected, the degree of agreement increases as more data is available per transcript (e.g. to Pearson r 0.81, 0.94 at median expression quantile and up, Figure S1I). This suggests that to the degree that a single measurement is worse than a full timecourse estimate, it can mostly be explained by decreased data availability, rather than the dynamic aspect of the time course.

Next, to verify that the differences between the single measurement estimates and time course estimates were not driven by the extra free parameter per transcript (error rate), we plot the transcript specific fitted error rate and the ratio between the degradation rate fitted with the time course and a 10' single measurement (Figure S1J). We could not find a correlation, suggesting that transcript-specific error rates were not necessary for a better fit. Importantly, 98.3% of fitted transcript error rates were below 0.011, compared to a conversion rate of ~0.11, making the differences between error rates negligible in virtually all cases (Figure S1J).

Finally, as the fitted half-lives depend on global parameters (growth rate, labeling time) that are not directly estimated from the data, we wanted to examine their effects on individual half-life fit. This amounts to probing equation 1 with respect to γ and t. This analysis (Figure S1L) revealed that for most transcripts, a 2-fold error in growth rate estimate is insignificant (at least in wildtype conditions), as the active degradation is much faster than dilution. However, if the effective labeling time is different from the presumed labeling time (e.g. if there is an initial delay in 4tU incorporation, or a delay until molecules are observed due to polyadenylation) then the estimates of most transcripts will be offset by a constant.

Modeling transcript-specific xrn1 knockout effects

We tried to explain the log-fold changes to RNA level, degradation rates, and production rates observed between the wildtype and Xrn1 deletion strain at a steady-state using various feature sets and models.

We consider several basic features: ORF length, ORF GC content, 5' GC content, 3' GC content, and 3' UTR motif score(9). On top of these ("base"), we add additional features - translation efficiency (10), and various measures derived from the tRNA translation efficiency index (11). On top of these, we used DRIMust(12) to look for significantly occurring motifs in transcripts that are extreme in either response

(41 such sequences were found, mostly in ORFs and 3'UTRs) and considered their occurrences as additional features (termed "DM" in figure S2). Aside from these we also used a heuristic to select kmer-counts (1-7mers) as features by calculating correlations to data, sampling and using a sparse linear model (LASSO) as a way to assess feature importance. This was repeated on random sets of kmers until convergence to a small set of informative features. Kmers were calculated separately for each ORF, 200 bp upstream of ORF (5UTR), and 200 bp downstream of ORF (3UTR). In figure S2H "FS" indicates all of these features were used simultaneously for prediction. Subsets of these features were provided to a LASSO (or a random forest) procedure trying to regress the given features against the target vector (logFC in degradation/production/RNA levels for xrn1 KO relative to WT, or log levels of KO or WT). Results for various optimal models are shown in Figure S2G. Specific feature combination performance is shown for the differences between KO and WT in Figure S2H, and informative kmers are shown in S2I.

First-order model predictions when degradation is reduced but production is not

Assuming a first-order model, the equation governing mRNA levels, R(t), following parameter changes is (neglecting the effects of growth):

$$R(t) = \frac{\pi}{\delta} + (\frac{\pi'}{\delta'} - \frac{\pi}{\delta})(1 - e^{-t\delta'})$$

Where the primed parameters (') are the new ones. Notably, at R(0) the value is the old steady-state and at t=inf the value is the new steady state. Notably, if the production rate doesn't change at t=0 then π simply scales the entire equation, i.e. if we consider the changes relative to t=0 we will remove any effects of the production rate from the signal. (red/purple, on the right-hand side, below). Therefore, we consider the changes from t=0 by calculating the difference, i.e.:

$$dR(t) = R(t) - \frac{\pi}{\delta} = (\frac{\pi'}{\delta'} - \frac{\pi}{\delta})(1 - e^{-t\delta'})$$

Or in terms of the slope:

$$S = \frac{1}{t}dR(t) = \frac{1}{t}(\frac{\pi'}{\delta'} - \frac{\pi}{\delta})(1 - e^{-t\delta'})$$

Now if we assume production is constant, we have:

$$\log S = \log \pi + \log \frac{1}{t}(\frac{1}{\delta'} - \frac{1}{\delta})(1 - e^{-t\delta'})$$

For relatively small new degradation rates (δ'), the right-hand side of the expression can be significantly simplified as

$$(\frac{1}{\delta'} - \frac{1}{\delta}) \approx \frac{1}{\delta'}$$

And as

$$(1 - e^{-t\delta'}) \approx \delta' t$$

Therefore:

$$\log S \approx \log \pi + \log(\frac{1}{t}\frac{1}{\delta'}\delta' t) = \log \pi$$

To illustrate this point, plotted below are the absolute, difference, and relative values of such responses when various parameters are changed (legend). They are grouped by their parameters. The case with the same production rate and initial degradation rate that changes to a variety of degradation rates is in green, the case with various production rates and a relatively short new half-life is in purple, and the case with various production rates and a relatively long new half-life is in red - where there's the highest correlation of slate slope to the production rate.

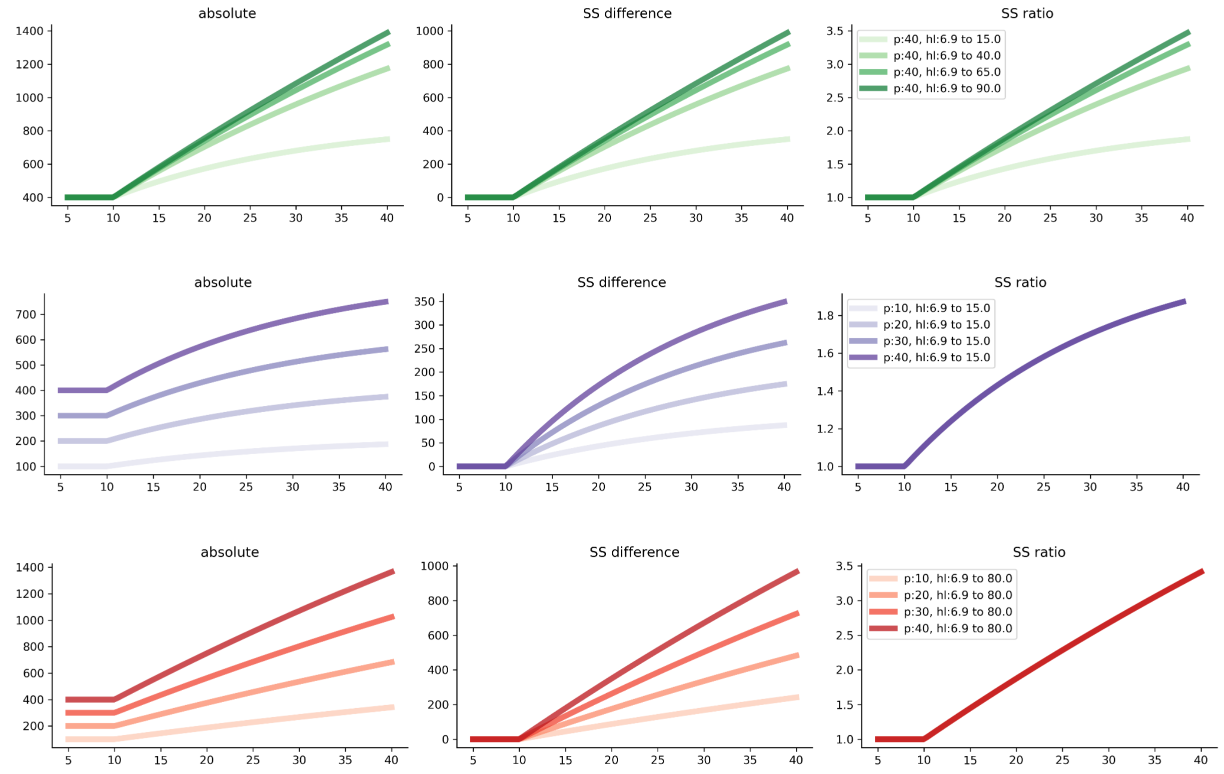

Not1 sensitive transcripts

mRNA trajectories were fitted with a pulse model, and the maximum up to t = 120 was interpolated from the fitted model. Transcripts whose Not1 maximum value was larger than Xrn1 max value by at least a standard deviation of the wildtype maximum value (used as a null model) were considered "Not1 sensitive".

Cell cycle signature analysis

Even though the population of cells we studied was unsynchronized, the time scale of 60-90 minutes was in the order of the yeast cell cycle (~90'). We reasoned that if cells adapt to the absence of Xrn1, and this adaptation is linked to the cell cycle, there might be a transient cell-cycle signal following Xrn1 depletion that dissipates as cells adapt. To test this prediction, we used our measurements following Xrn1 depletion in an unsynchronized population and specifically examined the changes in transcription. While the

cell-cycle gene sets start like all other genes, we observed a faster and more pronounced transcription reduction in cycling G1 and S genes compared to other cell cycle groups or the rest of the transcriptome (Kolmogorov-Smirnov q 10^{-2}-10^{-18}, Figure 5I, S6A-B). As expected from an adaptive response, this signature dissipates as the cells arrive at their new steady-state, and is absent in the knockout (Figure S6A). Following this observation, we monitored the cell-cycle distribution of an unsynchronized population following Xrn1 depletion (Figure S6D), and observed gross changes in DNA content distribution only after three hours. Thus, the earlier and subtle reduction in the transcription of G1 and S genes cannot be explained by changes to the distribution of cells along the cell cycle.

To verify that this cell-cycle signature is not an artifact, we repeated the analysis in time courses for various knockdowns and found no cell-cycle signal in most cases (Figure 5I, S6B). A notable exception is a depletion of the essential chromatin remodeler Sth1, where we observed an opposite signature, i.e. the transcription of G1/S genes decreases later than other genes (Figures S6B-C), perhaps related to reports that interference with Sth1 causes a G2/M arrest (13–15). Since the G1 and S sets were the only ones to show this effect (in 5'-3' factors and Sth1), we were worried that this set of genes has some property that makes it more susceptible in this analysis. We, therefore, selected sets of genes with similar half-life and expression levels and repeated the analysis, but found no significant hits (Figures S6E-F), excluding the possibility of an analysis artifact emanating from extreme mRNA levels or degradation rates.

We conclude that the reduction in the transcription adaptation response in arrested cells, and the unique and transient cell cycle signature upon Xrn1 depletion in an unsynchronized population point to a link between the cell cycle and the transcription adaptation response.

Xrn1 depletion in a G1-arrested population

To further explore a potential role for the cell cycle in the transcription adaptation response, we performed cDTA-seq on samples from an Xrn1 depletion time-course in cycling and G1-arrested cultures (Figure S7A). When we compared the response to Xrn1 depletion in arrested/cycling cells, the accumulation of mRNA is evident and highly significant in both cycling and arrested cells (median increase of 46.3% and 22.9% increase respectively after 60 minutes, Fig S7B). Furthermore, changes to mRNA through the time course are similar between cycling and arrested cells (Figures S7C-D), indicating that the depletion of Xrn1 induces the same immediate response even though arrested cells begin at a different basal state (Figures S7E-F). However, when we examined the transcription adaptation response, we observed only a minimal reduction in transcription in arrested cells compared to cycling cells after 90 minutes (Figures S7B, D).

To exclude the possibility that we are unable to reliably measure a decrease in transcription relative to the initial arrested state, we depleted Med14 - an essential component of the mediator (16) - which causes a significant transcription reduction even in arrested cells (Figure S7F).

Having verified our ability to measure transcriptional shutdown in arrested conditions, we asked whether the lack of response could be explained by delayed dynamics due to the different basal states. More

specifically, since arrested cells are larger, but contain less mRNA (figure S7E), which also accumulates slower (Fig S7B), it is possible that in a longer experiment the depletion of Xrn1 would eventually lead to a decrease in transcription even in arrested cells. However, we were limited in our ability to probe the response to Xrn1 depletion for long durations in arrested cells due to their disrupted physiology and eventual escape from induced G1-arrest. As an alternative, we reasoned that if there is a delay in the transcriptional response in arrested cells, then we expect the signature of recently-transcribed mRNA to temporally diverge from the signature of a cycling population responding to Xrn1 depletion. More specifically - we expect that later time points in an arrested population will be more similar to earlier time points in a cycling population. To try and address this hypothesis, we used a different experiment as a reference point, and compared the changes in recently-transcribed mRNA following Xrn1 depletion to the same profile 90' after Xrn1 depletion in cycling/arrested cells (figure S7H). However, we found that both profiles (arrested/cycling after 90 minutes) were most correlated to the 90-120 timepoints from the other experiment. If anything, it seems that the 90' arrested sample was more correlated to the 120' reference (cycling) time point, which is the opposite of the expectation from a delayed response. We conclude that there is no evidence that the transcriptional response is delayed in arrested cells, but that more experimentation is required to provide more direct evidence that the transcriptional response is ablated in arrested cells.

To summarize, there are two lines of evidence pointing to the cell cycle as a potential regulator of mRNA homeostasis. First, we identified a clear G1- and S-phase signature in unsynchronized populations, following the depletion of all 5'-3' degradation factors. Secondly, when we depleted Xrn1 in G1-arrested cells we observed only a modest decrease in transcription. Even though both lines of evidence are circumstantial, they point to a potential role for the cell cycle in the observed feedback. The observed G1 and S signature in the unsynchronized population and the delayed reduction in transcription, suggest a sensing mechanism that is triggered in a specific stage of the cell cycle after some time has passed since Xrn1 depletion (Figure S7I).

Supplementary References

1. Love,M.I., Huber,W. and Anders,S. (2014) Moderated estimation of fold change and dispersion for RNA-seq data with DESeq2. *Genome Biology*, **15**.

2. Vallat,R. (2018) Pingouin: statistics in Python. *Journal of Open Source Software*, **3**, 1026.

3. Lu,A.X., Zarin,T., Hsu,I.S. and Moses,A.M. (2019) YeastSpotter: accurate and parameter-free web segmentation for microscopy images of yeast cells. *Bioinformatics*, **35**, 4525–4527.

4. Jonas,F., Soifer,I. and Barkai,N. (2018) A Visual Framework for Classifying Determinants of Cell Size. *Cell Rep.*, **25**, 3519–3529.e2.

5. Jorgensen,P. (2002) Systematic Identification of Pathways That Couple Cell Growth and Division in Yeast. *Science*, **297**, 395–400.

6. Zhang,J., Schneider,C., Ottmers,L., Rodriguez,R., Day,A., Markwardt,J. and Schneider,B.L. (2002) Genomic Scale Mutant Hunt Identifies Cell Size Homeostasis Genes in S. cerevisiae. *Current Biology*, **12**, 1992–2001.

7. Klein-Brill,A., Joseph-Strauss,D., Appleboim,A. and Friedman,N. (2019) Dynamics of Chromatin and Transcription during Transient Depletion of the RSC Chromatin Remodeling Complex. *Cell Reports*, **26**, 279–292.e5.

8. Santos,A., Wernersson,R. and Jensen,L.J. (2015) Cyclebase 3.0: a multi-organism database on cell-cycle regulation and phenotypes. *Nucleic Acids Res.*, **43**, D1140–4.

9. Cheng,J., Maier,K.C., Avsec,Ž., Rus,P. and Gagneur,J. (2017) -regulatory elements explain most of the mRNA stability variation across genes in yeast. *RNA*, **23**, 1648–1659.

10. Cheng,Z., Mugler,C.F., Keskin,A., Hodapp,S., Chan,L.Y.-L., Weis,K., Mertins,P., Regev,A., Jovanovic,M. and Brar,G.A. (2019) Small and Large Ribosomal Subunit Deficiencies Lead to Distinct Gene Expression Signatures that Reflect Cellular Growth Rate. *Mol. Cell*, **73**, 36–47.e10.

11. Sabi,R., Volvovitch Daniel,R. and Tuller,T. (2017) stAIcalc: tRNA adaptation index calculator based on species-specific weights. *Bioinformatics*, **33**, 589–591.

12. Leibovich,L., Paz,I., Yakhini,Z. and Mandel-Gutfreund,Y. (2013) DRIMust: a web server for discovering rank imbalanced motifs using suffix trees. *Nucleic Acids Res.*, **41**, W174–9.

13. Cao,Y., Cairns,B.R., Kornberg,R.D. and Laurent,B.C. (1997) Sfh1p, a component of a novel chromatin-remodeling complex, is required for cell cycle progression. *Mol. Cell. Biol.*, **17**, 3323–3334.

14. Du,J., Nasir,I., Benton,B.K., Kladde,M.P. and Laurent,B.C. (1998) Sth1p, a Saccharomyces cerevisiae Snf2p/Swi2p Homolog, Is an Essential ATPase in RSC and Differs From Snf/Swi in Its Interactions With Histones and Chromatin-Associated Proteins. *Genetics*, **150**, 987–1005.

15. Angus-Hill,M.L., Schlichter,A., Roberts,D., Erdjument-Bromage,H., Tempst,P. and Cairns,B.R. (2001) A Rsc3/Rsc30 zinc cluster dimer reveals novel roles for the chromatin remodeler RSC in gene expression and cell cycle control. *Mol. Cell*, **7**, 741–751.

16. Warfield,L., Ramachandran,S., Baptista,T., Devys,D., Tora,L. and Hahn,S. (2017) Transcription of Nearly All Yeast RNA Polymerase II-Transcribed Genes Is Dependent on Transcription Factor TFIID. *Molecular Cell*, **68**, 118–129.e5.
